# Supplementary material for: Exercise promotes the functional integration of human stem cell-derived neural grafts in a rodent model of Parkinson’s disease
Source: Stem Cell Reports. 2025 Apr 24;20(5):102480. doi: 10.1016/j.stemcr.2025.102480 (PMC12143144; doi:10.1016/j.stemcr.2025.102480)
Supplement: Document S1. Figures S1–S5 and Table S1 [file mmc1.pdf]

Stem Cell Reports, Volume *20*

Supplemental Information

Exercise promotes the functional integration of human stem cell-derived neural grafts in a rodent model of Parkinson's disease

Niamh Moriarty, Tyra D. Fraser, Cameron P.J. Hunt, Georgia Eleftheriou, Jessica A. Kauhausen, Lachlan H. Thompson, and Clare L. Parish

SUPPLEMENTARY DATA

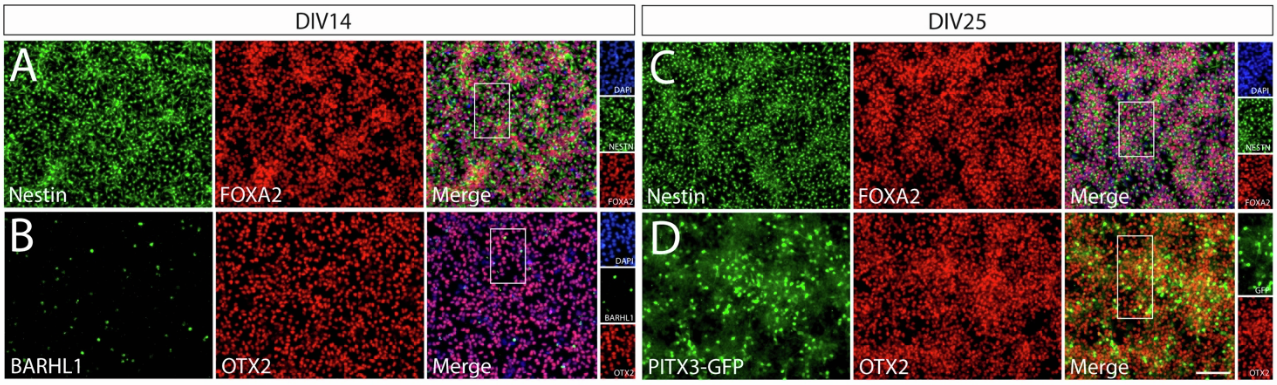

Supplementary Figure 1: Ventral midbrain dopaminergic neurons differentiated from a human induced pluripotent stem cell line expressing enhanced green fluorescent protein under the PITX3 promoter, PITX3-GFP. Photomicrographs showing high FOXA2 and OTX2 expression at D14 (***A,B***). Low numbers of BARHL1+ cells, indicative of off-target rostral progenitors, further validate VM differentiation efficacy (***B***). Ventral midbrain dopamine neuron differentiation was confirmed at D25 by Nestin, FOXA2, PITX3-GFP, and OTX2 expression (***C, D).*** Day (D). Scale bar: 200μm (***A-D***).

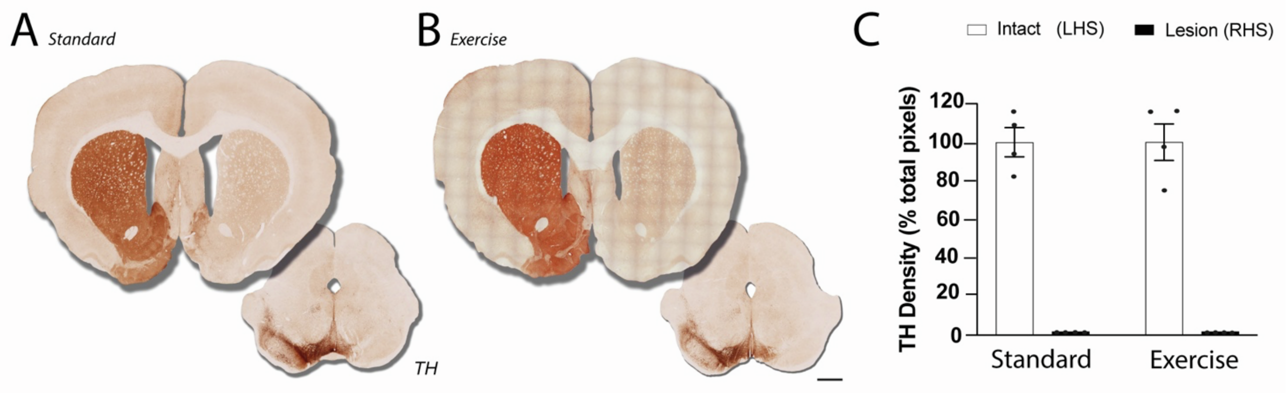

Supplementary Figure 2: Exercise had no impact on the residual host dopaminergic system. Representative images of TH+ immunohistochemistry confirm unilateral 6OHDA lesions in standard (***A***) and exercise (***B***) conditions at 24 weeks. Quantitative analysis of TH+ fibers confirmed that exercise had no impact on the density of dopamine fibers in the host dorsolateral striatum (**C**). Tyrosine Hydroxylase (TH). Data are Mean ± SEM. n=4/group. Scale bar: 1mm (***A, B***).

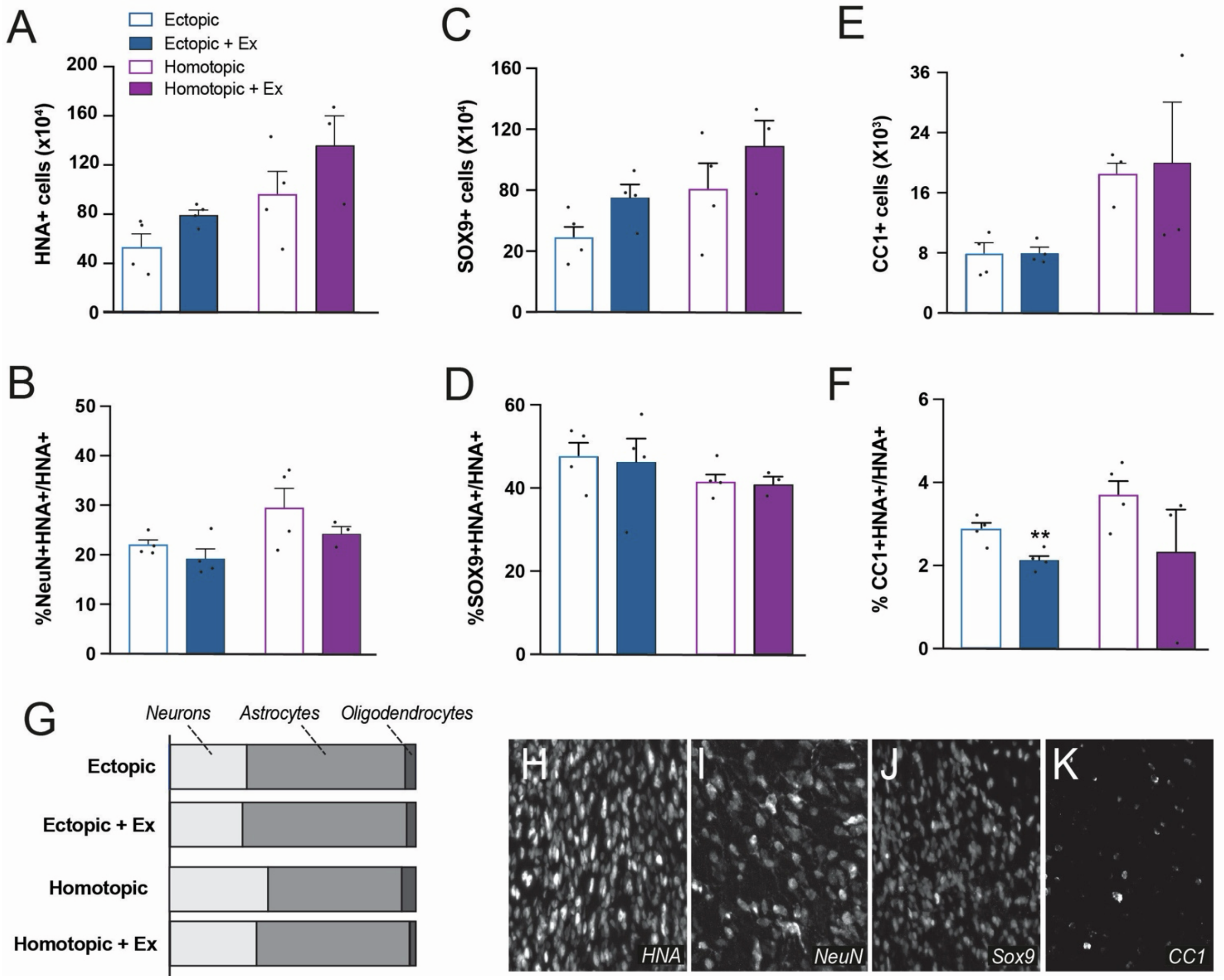

Supplementary Figure 3: Quantification of graft composition revealed that exercise had no significant impact on the total number of HNA+ human cells (**A**), their proportion of NeuN+ neurons (**B**), number or proportion of SOX9+ astrocytes (**C, D)**, or CC1+ oligodendrocytes numbers (**E,F**). Only the proportion of CC1+ cells was reduced in ectopic grafts after exercise (**F**). Representation of the proportion of neurons, astrocytes and oligodendrocytes within hPSC-derived grafts (**G**). Representative images depicting HNA (**H**), NeuN (**I**), SOX9 (**J**) and CC1 (**K**) staining within grafts. Human Nuclear Antigen (HNA). Scale bars: 200um (H-K). Data are Mean ± SEM. ***p*<0.01 vs standard. n=4/group.

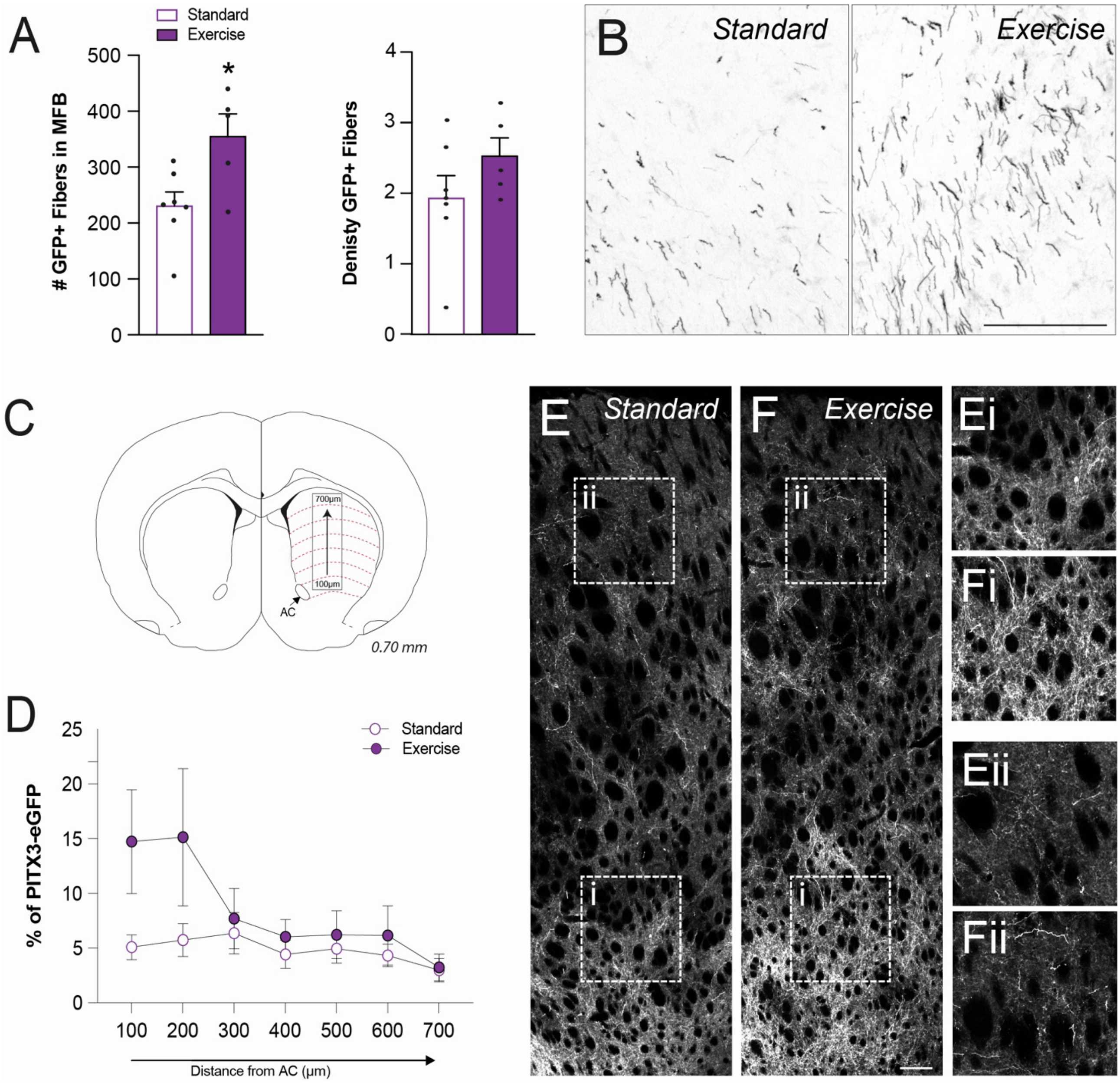

Supplementary Figure 4: Exercise increases dopamine fiber growth along the nigrostriatal pathway. Quantitative analysis revealed a significant increase in the number of fibers transcending through the medial forebrain bundle in animals undergoing an exercise regime (*A*). Photomicrographs showing dopamine fibers in the medial forebrain bundle (*B*). Schematic showing sampling sites along the ventral-dorsal tier of the striatum, 100-700μm from the Anterior Commissure (C). Exercise increased GFP+ fiber density in the ventral striatum, where dopamine fibers emanate from the medial forebrain bundle, however failed to enhance innervation in dorsal tiers (*D*). Representative images showing innervation patterns across the ventral – dorsal striatum under standard (*E*) and exercise (*F*) conditions. High magnification images show GFP+ fibers in the ventral (*Ei, Fi*) and dorsal (*Eii, Fii*) striatum. Anterior Commissure (AC). Data are Mean ± SEM. *$p<0.05$ vs standard. n=5-6/group. Scale bar: 200μm (*B, E, F*).

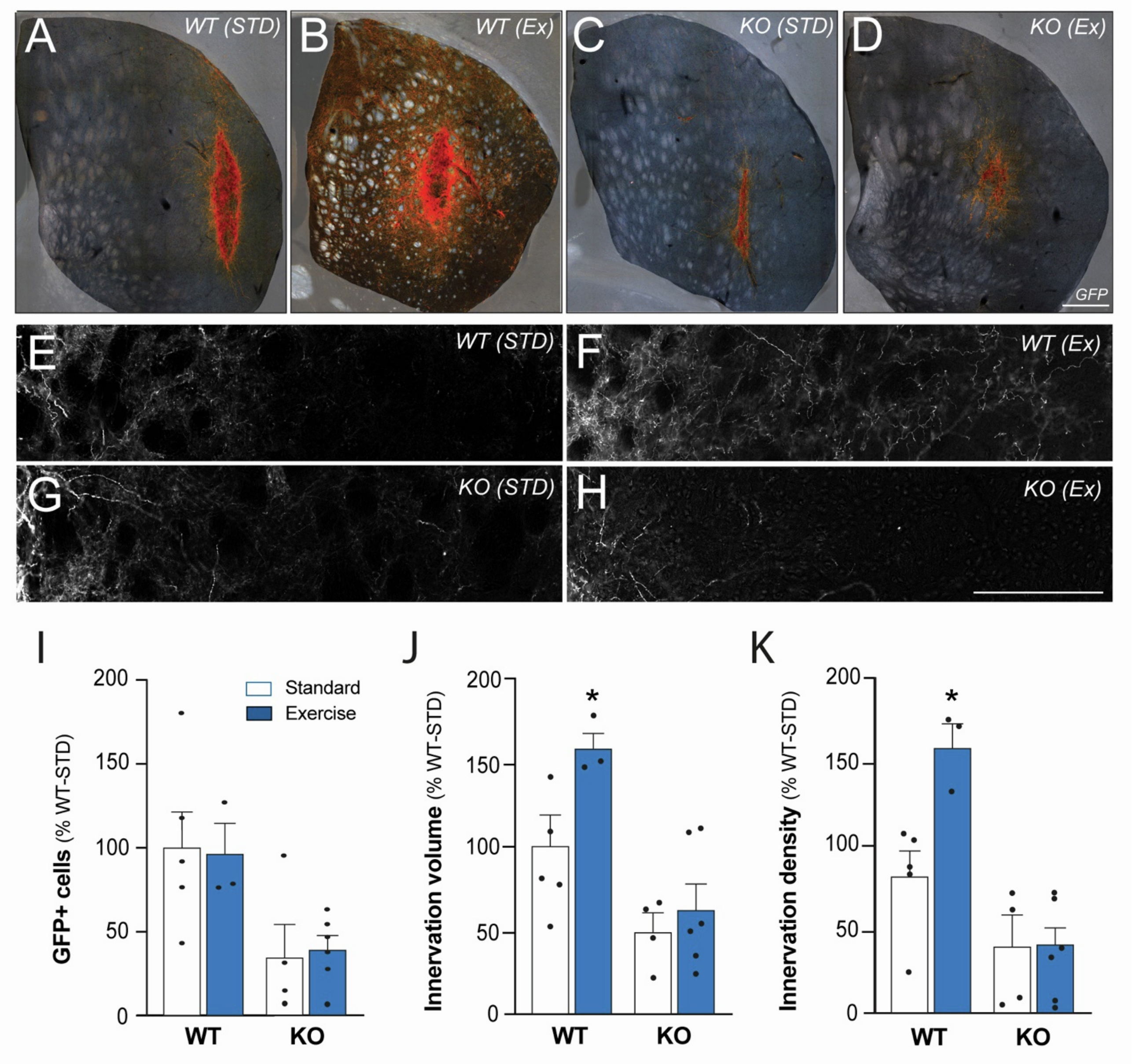

Supplementary Figure 5: Exercise promotes plasticity in WT, but not GDNF-KO mice. Representative images of fetal-derived dopaminergic grafts in the striatum of WT and GDNF-KO mice housed under standard (*A, C, E, F*) or exercise (*B, D, G, H*) conditions. Exercise had no impact on the number of GFP+ cells in WT or GDNF-KO mice (*I*). Exercise has a significant impact on the graft-derived innervation volume (J) and density within the striatum (K) in WT but not KO mice. Exercise (Ex); Knockout (KO); Standard (STD); Wildtype (WT). Data are Mean ± SEM. *p<0.05 vs standard. n=6/group. Scale bar: 1mm (*A-D*).

BARHL1	Rabbit	Novus Biologicals	Cat# NBP1-86513	1:200
BDNF	Rabbit	Abcam	Cat# ab46176	1:2000
Calbindin-C28K	Mouse	Swant	Cat# 300	1:1000
DAPI	-	Sigma Aldrich	Cat# D8417	1:5000
ERK	Rabbit	Cell Signaling Tech	Cat# 91025	1:300
CC1	Mouse	Abcam	Cat# ab16794	1:200
cFOS	Goat	Santa Cruz	Cat# sc-52	1:1000
FOXA2	Goat	Santa Cruz	Cat# sc-6554	1:200
GDNF	Rabbit	Invitrogen	Cat# PA5-89957	1:2000
Green fluorescent protein (GFP)	Chicken	Abcam	Cat# ab13970	1:1000
GFP	Rabbit	Abcam	Cat# ab290	1:1000
GIRK2	Rabbit	Abcam	Cat# ab65096	1:500
Nestin	Mouse	R&D Systems	Cat# MAB353	1:1000
NeuN	Mouse	Millipore	Cat# MAB377	1:1500
OTX2	Goat	R&D Systems	Cat# AF1979	1:500
pERK	Rabbit	Cell Signaling Tech	Cat# A1065	1:300
RECA-1	Mouse	Abd Serotec	Cat# MCA970R	1:2000
SOX9	Rabbit	Abcam	Cat# ab185966	1:500
Human Synaptophysin (hSYP)	Mouse	Enzo Life Sciences	Cat# ADI-905-782-100	1:200
PITX3	Goat	Santa Cruz	Cat# sc-19307	1:200
Tyrosine Hydroxylase (TH)	Rabbit	Pel-freeze	Cat# P40101-0	1:1000
TH	Sheep	Pelfreeze	Cat# P60101-0	1:800

Supplementary Table 1: List of antibodies and dilutions.
