# Supplementary material for: Exercise promotes the functional integration of human stem cell-derived neural grafts in a rodent model of Parkinson’s disease
Source: Stem Cell Reports. 2025 Apr 24;20(5):102480. doi: 10.1016/j.stemcr.2025.102480 (PMC12143144; doi:10.1016/j.stemcr.2025.102480)
Supplement: Document S2. Article plus supplemental information [file mmc2.pdf]

Stem Cell Reports

Article

OPEN ACCESS

Exercise promotes the functional integration of human stem cell-derived neural grafts in a rodent model of Parkinson's disease

Niamh Moriarty,[1] Tyra D. Fraser,[1] Cameron P.J. Hunt,[1] Georgia Eleftheriou,[1] Jessica A. Kauhausen,[1] Lachlan H. Thompson,[1,2,3,*] and Clare L. Parish[1,3,4,*]

[1]The Florey Institute of Neuroscience and Mental Health, The University of Melbourne, Parkville, Victoria, Australia

[2]Charles Perkins Institute, The University of Sydney, Sydney, NSW, Australia

[3]These authors contributed equally

[4]Lead contact

*Correspondence: lachlan.thompson@sydney.edu.au (L.H.T.), cparish@unimelb.edu.au (C.L.P.)

https://doi.org/10.1016/j.stemcr.2025.102480

SUMMARY

Human pluripotent stem cell (hPSC)-derived dopamine neurons can functionally integrate and reverse motor symptoms in Parkinson's disease models, motivating current clinical trials. However, dopamine neuron proportions remain low and their plasticity inferior to fetal tissue grafts. Evidence shows exercise can enhance neuron survival and plasticity, warranting investigation for hPSC-derived neural grafts. We show voluntary exercise (wheel running) significantly increases graft plasticity, accelerating motor recovery in animals receiving ectopic, but not homotopic, placed grafts, suggestive of threshold requirements. Plasticity was accompanied by increased phosphorylated extracellular signal-regulated kinase (ERK+) cells in the graft (and host), reflective of mitogen-activated protein kinase (MAPK)-ERK signaling, a downstream target of glial cell-derived neurotrophic factor (GDNF) and brain-derived neurotrophic factor (BDNF), proteins that were also elevated. Verifying improved graft integration was the increase in cFos+ postsynaptic striatal neurons. These findings have direct implications for the adoption of physical therapy-based approaches to enhance neural transplantation outcomes in future Parkinson's disease clinical trials.

INTRODUCTION

The generation of defined neuronal populations from human pluripotent stem cells (hPSCs) has instigated an exciting new era for brain repair. Preclinical data demonstrate that hPSC-derived neural grafts, enriched with dopamine (DA) neurons, can structurally and functionally integrate in the brain of Parkinson's Disease (PD) models to reverse motor deficits, motivating current clinical trials (Barker et al., 2017). While providing essential proof of concept, these preclinical trials show low DA neuron proportions and suboptimal plasticity compared to fetal-derived ventral midbrain (VM) grafts that may impact functional efficacy (Grealish et al., 2014).

An area of preclinical and clinical research showing promise in influencing neuronal survival and plasticity, while remaining non-invasive, and therefore of likely rapid translation, is exercise. The benefits of exercise on disease progression, measured in part using the Unified Parkinson's Disease Rating Scale and perceived quality of life, are well reported (Mak et al., 2017), including a 2021 Cochrane systematic review (Ernst et al., 2023). However, only recently have we begun to understand the mechanisms underpinning these benefits. Preclinical studies reported increased neurotrophin levels, regulation of medium spiny neurons including recovery of dendritic spine density (Toy et al., 2014), rescue of corticostriatal long-term potentiation, and slowing of toxic a-synuclein spread in the brain (Bastioli et al., 2022; da Silva et al., 2016; Marino et al., 2023; Zhou et al., 2017), while clinical studies report greater modulation of neuroinflammation, upregulation of DA transporter expression (de Laat et al., 2024; Malczynska-Sims et al., 2022), and elevated DA release (Johansson et al., 2022).

While the ability to slow disease progression is significant, this strategy fails to reverse existing motor symptoms. In the context of neural transplantation, experience-driven plasticity, via environmental enrichment and exercise, has been shown to enhance survival and integration of new neurons. First demonstrated by Mayer et al., in a striatal lesion model, amelioration of motor deficits was accelerated in animals receiving neural grafts and post-operative motor testing, concluding that function could be improved if animals "learn to use" their transplant through relevant experience (Mayer et al., 1992). Since then, others have demonstrated benefits for fetal neural grafts in animal models of Huntington's disease, stroke, and spinal cord injury (Brasted et al., 1999a, 1999b, 2000; 2000; Hwang et al., 2014; Tashiro et al., 2016; Wu et al., 2022).

For PD, studies describing the impact of environmental cues on graft outcomes are notably few. In 2000, Dobrossy et al. were the first to report increased plasticity of fetal-derived DA neurons in the intact brain (Dobrossy et al., 2000), followed recently in PD rats in response to forced exercise (Torikoshi et al., 2020). What remains to be

© 2025 The Author(s). Published by Elsevier Inc. on behalf of International Society for Stem Cell Research. This is an open access article under the CC BY license (http://creativecommons.org/licenses/by/4.0/).

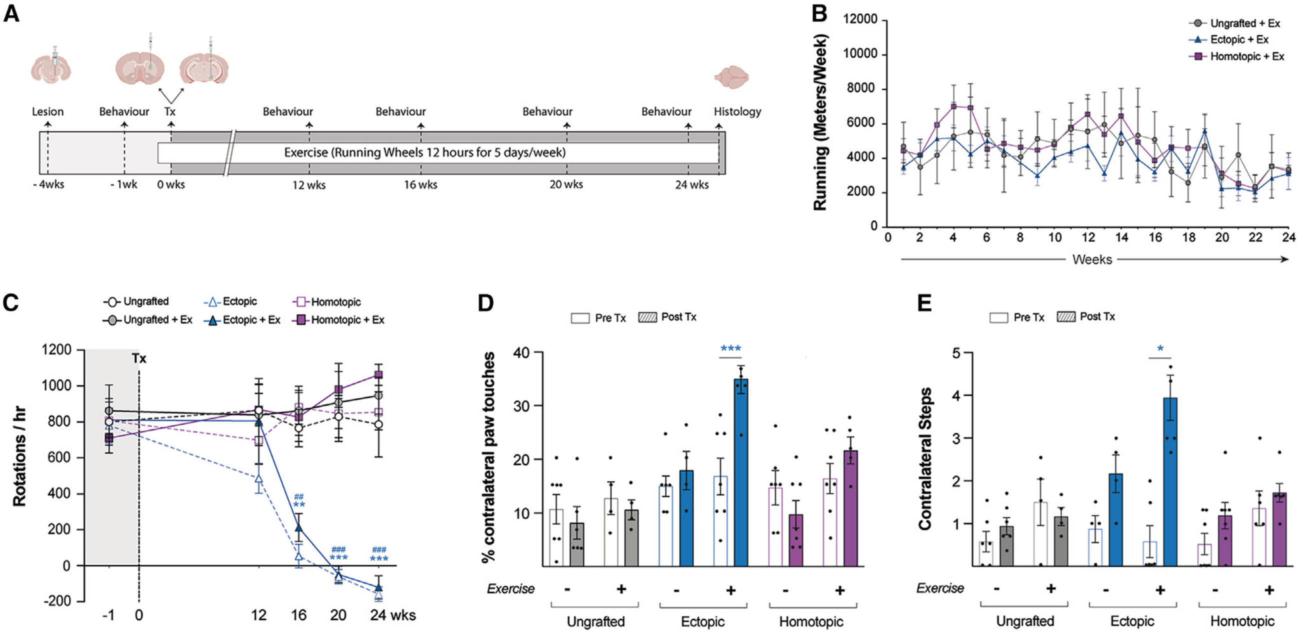

Figure 1. Exercise significantly enhances the functional integration of ectopically placed DA neurons

(A) Schematic depicting *in vivo* study design highlighting the ectopic (intrastriatal) or homotopic (intranigral) grafting of hPSC-derived VM progenitors prior to an exercise regime consisting of voluntary running (12 h/5 days/week).

(B) Animals maintained running over the study duration.

(C) By 24 weeks post-grafting, animals receiving homotopic grafts ± exercise failed to restore motor function in the amphetamine rotational test, while animals with ectopic grafts ± exercise showed full recovery ($p < 0.0001$).

(D and E) Only animals within the ectopic + exercise group exhibited recovery in the (D) cylinder ($p = 0.0058$) and (E) adjusted stepping ($p = 0.0004$) tests at 24 weeks.

Abbreviations: Ex, exercise; Tx, transplant. Data are Mean ± SEM. n = 4–7/group. $^{**}p < 0.01$, $^{***}p < 0.001$ vs. ungrafted. $^{\#\#}p < 0.01$, $^{\#\#\#}p < 0.001$ vs. ungrafted + exercise. $^{*}p < 0.05$, $^{**}p < 0.01$ vs. pre-Tx (D and E).

determined is the potential benefit of exercise on the functional outcomes of neural grafts in PD models, and whether such modest interventions may also impact homotopic grafts to promote reconstruction of midbrain circuitry. Finally, as the field embarks on clinical trials for PD using a more standardized, bioavailable, and less ethically contentious donor cell source for neural grafting, these assessments need to be made in the context of hPSC-derived DA grafts.

RESULTS

Exercise promotes the functional integration of ectopic, but not homotopically placed, hPSC-derived VM grafts

VM DA progenitors were differentiated from a human induced pluripotent stem cell (iPSC) line expressing enhanced green fluorescent protein under the PITX3 promoter, PITX3-GFP (Moriarty et al., 2022b), as previously described (Gantner et al., 2020a; Niclis et al., 2017). The use of the GFP reporter enabled selective tracking of DA neurons (expressing PITX3) *in vivo*. Differentiation efficacy was confirmed by high OTX2+ and FOXA2+ co-expression at day 14 (D14) *in vitro* (Figures S1A and S1B) and limited off-target PITX2, PAX6, or BARHL1+ cells (indicative of lateral, dorsal, and rostral populations) (Figure S1B, and data not shown). By D25, cells co-expression of Nestin and FOXA2 as well as PITX3-GFP and OTX2, demonstrating regional specification of VM progenitors and maturation into VM DA neurons (Figures S1C and S1D).

PITX3-GFP iPSC-derived VM progenitors (D19) were injected *ectopically* in the host striatum to assess their capacity to restore local circuitry or *homotopically* into the VM to reconstruct the nigrostriatal pathway (Figure 1A). All 6-hydroxydopamine (6OHDA) lesioned rats were assessed for amphetamine-induced rotational asymmetry. Only rats showing >6 rotations/min were included in the study and stratified across the following groups: 6OHDA lesions (ungrafted), ungrafted + exercise (Exercise), PITX3-GFP DA graft (Ectopic or Homotopic), or PITX3-GFP DA graft + exercise (Ectopic + Exercise or Homotopic + Exercise). Rats

underwent motor tests 1 week prior to, and at defined intervals post-grafting (Figure 1A). Rats in the Exercise groups had running wheel access during their 12 h dark cycle for 5 day/week and showed persistent running over the 24 week period, with no significant difference between the ungrafted and grafted exercise groups (Figure 1B).

Amphetamine-induced rotational asymmetry revealed stable motor deficits over 24 weeks in the ungrafted group (Figure 1C, white circles). In the absence of a graft, exercise had no impact on reversing motor dysfunction (Figure 1C, gray circles). Rats receiving a homotopic graft, with or without exercise, showed no improvement in rotational asymmetry by 24 weeks (Figure 1C, unfilled/filled purple squares, respectively). In contrast, rats receiving ectopic grafts (with or without exercise) showed complete recovery of rotational asymmetry by 24 weeks (Figure 1C, unfilled/filled blue triangles, respectively). In the cylinder and adjusted stepping tasks (assessments of spontaneous motor function), dysfunction was seen as reduced proportions of contralateral paw touches and the number of contralateral adjusted steps, respectively (gray bars, Figures 1D and 1E). All rats showed similar deficits prior to grafting (unfilled bars, Pre-Tx, Figures 1D and 1E). Recovery of asymmetrical paw touches and steps was only observed at 24 weeks in ectopic grafted rats with running wheel access (filled blue bars, Figures 1D and 1E).

Exercise promotes maturation of A9-like neurons in ectopic grafts, while homotopic grafts display increased proportions of non-DA neurons

Correlative with stable motor deficits observed in ungrafted, lesioned rats housed under standard conditions was the unilateral loss of midbrain TH+ DA neurons and associated striatal innervation at 24 weeks (Figures S2A and S2C). Importantly, exercise (in the absence of a graft) did not promote plasticity of residual host DA fibers in the striatum, eliminating any potentially confounding behavioral or histochemical analysis (Figure 1C gray circles; Figures S2B and S2C).

At 24 weeks after transplantation, all rats (±Exercise) showed surviving grafts, confirmed by GFP expression (Figures 2A–2D). GFP+ grafts were predominantly confined to the striatum (ectopic) or VM (homotopic). Consistent with our previous work, homotopic grafts were notably larger than ectopic (Moriarty et al., 2022a), with exercise baring no influence on volume at either site (Figure 2E). Exercise had no impact on GFP+ DA neuron numbers in ectopic or homotopic grafts (Figure 2F), and, reflective of increased graft volume, yet sustained GFP+ counts, rats receiving homotopic grafts (±exercise) showed a marked (̃50%) reduction in GFP cell density (GFP+cells/mm^3, data not shown).

To assess the ability of transplanted DA progenitors to mature into correctly specified A9 or A10-like DA subtypes, we assessed Calbindin (CALB) or GIRK2 immunoreactivity within GFP+ DA neurons, respectively (Figures 2G and 2H). Exercise significantly increased the proportion of GFP+GIRK2+ neurons, at the expense of GFP+CALB+ cells in ectopic grafts, yet had no impact on th eA9-or A10-like maturation of homotopic grafts (Figure 2G).

Given the increased homotopic verses ectopic graft volume, without corresponding change in GFP+ DA numbers, we performed a detailed assessment of graft composition (Figures 2 and S3). Total human nuclear antigen-labeled (HNA+) cells, NeuN+ neurons, SOX9+ astrocytes, and CC1+ oligodendrocytes within the graft commensurately increased in homotopic grafts (Figures 2I, 2L, and S3A), resulting in unchanged densities of these populations (Figures 2J, S3B–S3D, and S3F).

Interestingly, exercise had no effect on the number or proportion of DA and non-DA neurons or astrocytes at either graft site (Figures 2 and S3). A reduction in CC1+ oligodendrocyte proportion was observed yet only accounted for a small fraction of the transplants (Figure S3F).

Exercise promotes plasticity of dopaminergic neurons in ectopic grafts

Next, GFP+ immunolabeling was used to examine the plasticity of ectopic and homotopic grafts in response to exercise (Figures 3A–3D). Volumetric assessment of striatal area covered by GFP+ staining revealed significantly greater innervation by ectopic, compared to homotopic, grafts (Figure 3E), an effect that was exacerbated by exercise (Ectopic: 15.23 mm^3 ± 1.33; Ectopic + Exercise: 19.65 ± 0.96) (Figures 3B and 3E). Integration of dopaminergic neurons was further assessed by examination of GFP+ fiber density within defined striatal regions that underpin functions, inclusive of gross motor function (dorsolateral striatum) and more complex sensorimotor tasks (ventrolateral striatum), (Figures 3F–3I), as reported in preclinical (Chang et al., 1999; Mandel et al., 1990) and clinical fetal grafting studies (Piccini et al., 2005). Exercise significantly increased GFP+ DA fiber density from ectopic grafts within all striatal regions (dorsolateral: 1.9-fold; dorsomedial: 2.3-fold; dorsolateral 2.9-fold; dorsomedial: 3.1-fold) (Figures 3A–3D and 3F–3I). Only in the ventromedial striatum, where DA fibers emanate from the medial forebrain bundle, was a significant increase in GFP+ innervation observed in Homotopic + Exercise grafts (Figure 3I). This was complimented by an increase in GFP+ fibers traversing the medial forebrain bundle (Figure S4A), leading to increased ventral striatum innervation (100 μm dorsal of the anterior commissure; p = 0.058; Figures S4C–S4F), but not dorsal striatum—explaining the lack of motor improvements in these animals (Figures S4D–S4F and 3C).

With the impact of exercise showing only modest effects on homotopic grafts, we focused further assessments on

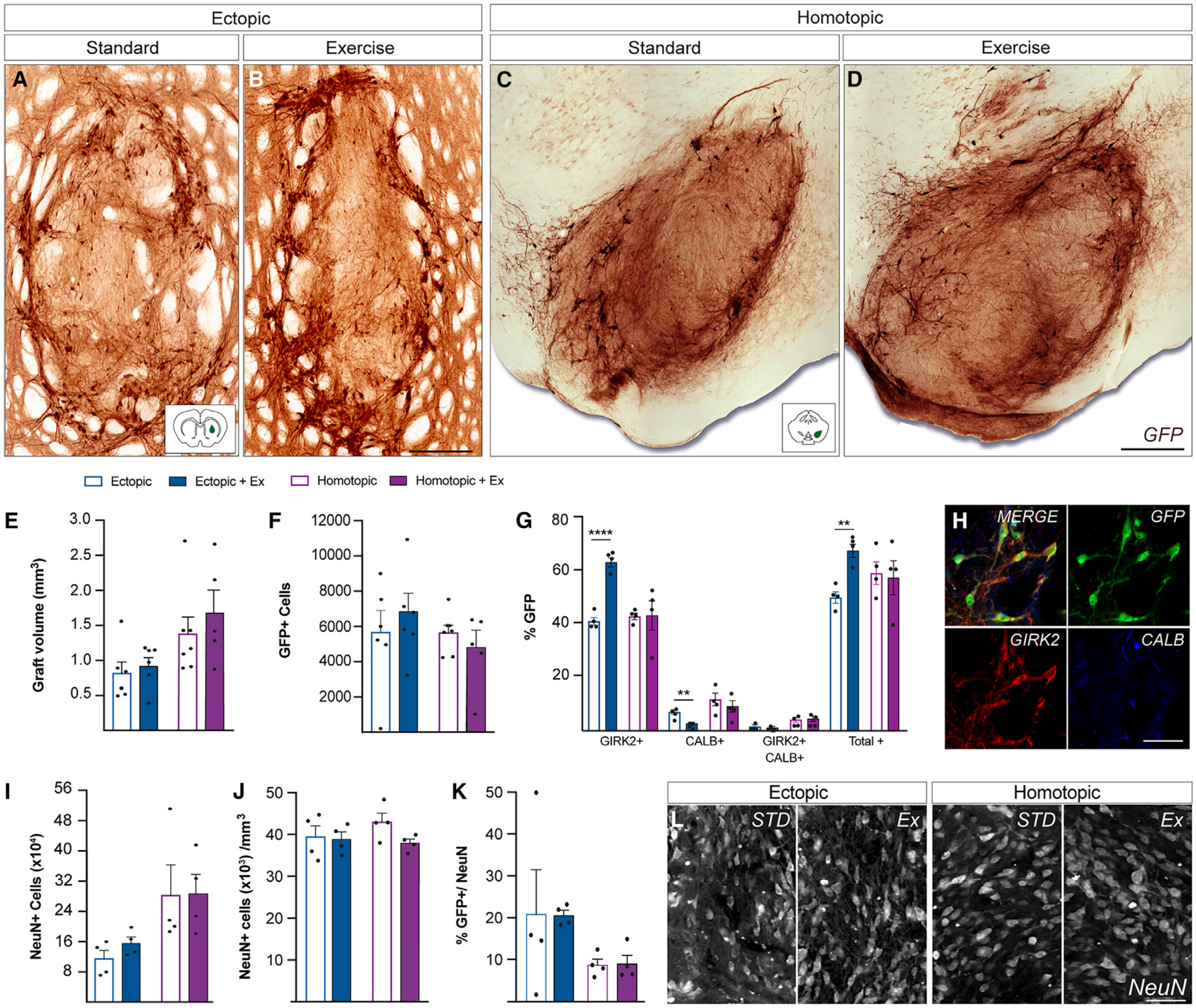

Figure 2. Exercise promotes the maturation of A9-like DA neurons within ectopically placed grafts

(A–F) Representative images show grafted GFP$^+$ DA neurons within the striatum (A and B) and midbrain (C and D). While homotopic grafts trended larger, exercise had no impact on graft volume (ectopic: $p = 0.632$, homotopic: $p = 0.461$, E) or GFP+ numbers (ectopic: $p = 0.485$, homotopic: $p = 0.411$, F).

(G) Exercise had no effect on DA phenotype maturation in homotopic grafts, while significantly increasing the proportion of A9 (GFP+GIRK2+; $p < 0.0001$) and decreaseing A10-like (GFP+CALB+; $p = 0.0047$) DA neurons within ectopic grafts.

(H–K) (H) Images showing DA neurons expressing GIRK2 (A9) and CALB (A10). Exercise had no significant impact on the number (ectopic: $p = 0.187$, homotopic: $p = 0.985$, I) or density (ectopic: $p = 0.879$, homotopic: $p = 0.072$, J) of NeuN+ neurons. However, given increased NeuN+ cells in homotopic grafts, GFP+ neuron proportion was reduced (K).

(L) Images show NeuN+ cells within ectopic and homotopic grafts.

Abbreviations: DA, dopamine; Ex, exercise; STD, standard. Data are Mean ± SEM. *$p < 0.05$, **$p < 0.01$, ***$p < 0.001$ vs. ectopic. $n = 4$–7/group. Scale bar: 1 mm (A–D) and 200 μm (H and L).

ectopic grafts. At intervals spanning 1.3 mm caudal to 1.2 mm rostral of the implantation site, the impact of exercise increasing DA fiber density across the striatal axis was sustained (Figures 3J and 3K). Regression analysis showed increased dorsolateral (and ventrolateral, not shown) striatal innervation positively correlated with distance ran/animal (Ectopic: $R^2 = 0.18$; Ectopic + Exercise: $R^2 = 0.66$; Figure 3L).

Relevant in the assessment of graft integration is not only connectivity of implanted DA neurons within the striatum,

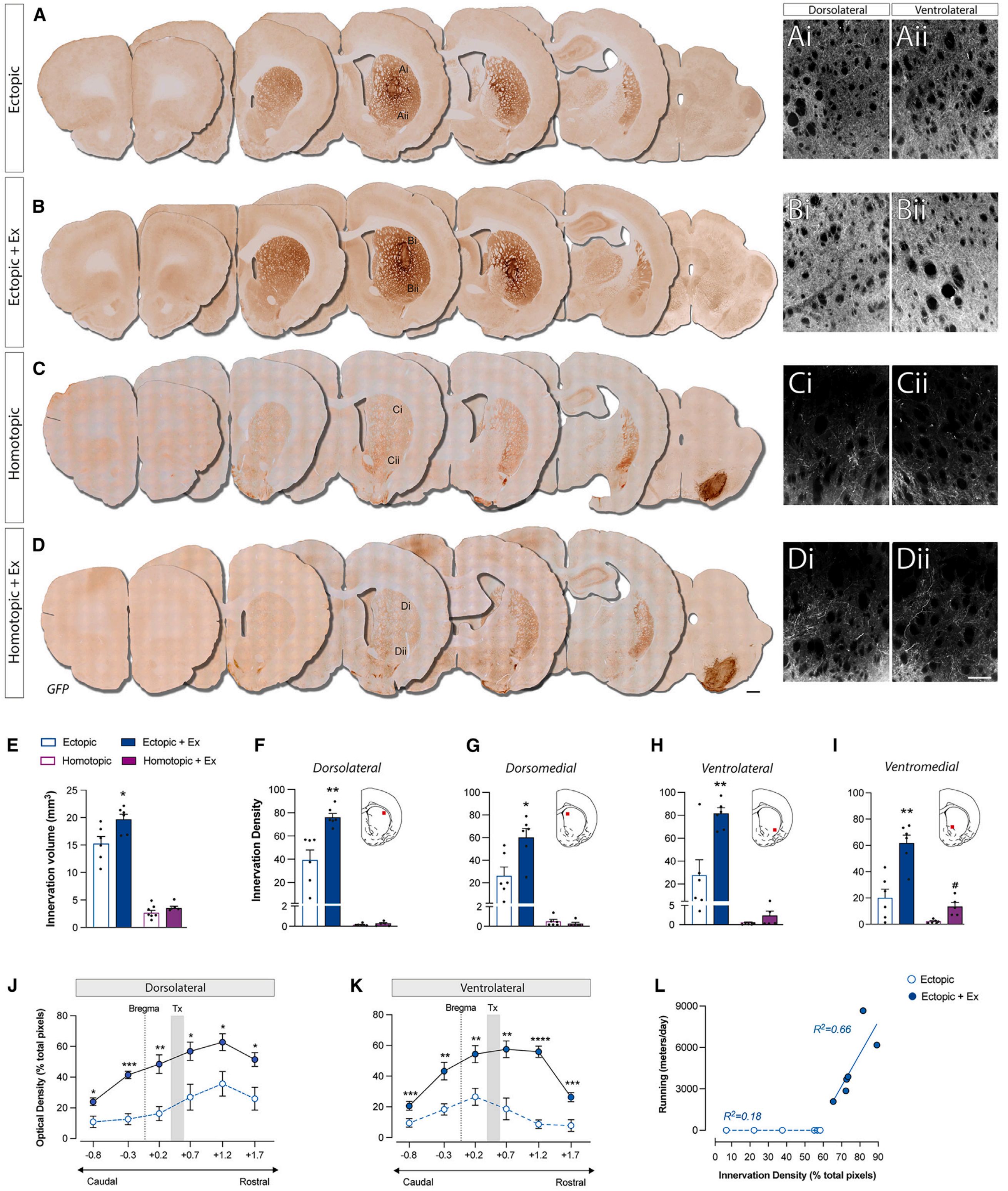

Figure 3. Exercise enhances plasticity of ectopically placed dopaminergic grafts

Coronal sections illustrate GFP+ DA fiber patterns of ectopic (A and B) and homotopic (C and D) grafts, with or without exercise. High-magnification images demonstrate the extent of innervation in the dorsolateral (Ai, Bi, Ci, and Di) and ventrolateral (Aii, Bii, Cii, and Dii)

(legend continued on next page)

but also other DA target nuclei as well as innervation emanating from non-DA neurons. Human specific synaptophysin (hSYP) and GFP immunostaining enabled for distinct characterization of graft-derived DA (GFP+hSYP+) and non-DA (GFP-hSYP+) terminals in the host (Figure 4A). hSYP+ staining revealed connectivity with other nuclei typically innervated by DA neurons including cortical areas, such as motor and perirhinal cortex (2 and 3, respectively, Figure 4B) as well as septum, thalamus, and amygdala, (4, 5, and 6, respectively, Figure 4B). While exercise significantly increased connectivity in the striatum, no significant change in extrastriatal targets were observed in response to exercise (Figures 4C–4E), highlighting that the positive impact of exercise on DA connectivity is selective to the striatum (the region involved in motor control). Assessment of non-DA innervation patterns (GFP-hSYP+) in the striatum and other target nuclei showed no change between animals housed in standard or exercise conditions, indicating that the effects of exercise in promoting plasticity were selective for DA neurons, and within key motor circuits (Figure 4D).

Exercise-induced graft integration drives enhanced postsynaptic host neuron activation through elevated ERK signaling

Acute upregulation of the intermediate early response gene, cFos, in medium spiny neurons (MSNs) of the striatum following amphetamine administration is modulated by DA release and subsequent receptor activity, providing an indirect measure of DA signaling (Cenci et al., 1992). To confirm increased GFP+ innervation in the host correlated with enhanced graft integration we assessed cFos labeling in the striatum of rats injected with amphetamine 1 h prior to perfusion. cFos+ activation was confirmed by CTIP2+/DARPP32+ co-expression (Figure 4F), with cFOS+ assessment performed in locations corresponding to GFP+ fiber density (dorsolateral, dorsomedial, ventrolateral, and ventromedial striatum; Figures 4G and 4H). As anticipated, lesioning reduced cFos expression in all areas of the striatum compared to the intact brain (Figure 4G, gray outline bar). Reflective of increased GFP fiber density, ectopic grafts (±Exercise) significantly increased cFos+ density to levels not different from the intact brain in the dorsolateral and dorsomedial striatum, yet only grafts in exercised animals showed significantly elevated cFos+ cells within the ventral tiers of the striatum, reflective of increased sensory motor circuit reconstruction (Figures 4G and 4H).

Exercise promotes angiogenesis within the host brain

Unlike fetal-derived VM grafts that have a subpopulation of vascular progenitors capable of establishing vessel networks that anastomose with the host brain, hPSC-derived neural progenitors intended for grafting lack such progenitors. These pluripotent stem cell-derived grafts rely on host angiogenesis and infiltration of new vessels into the grafted tissue. Previous studies, reporting on the impact of exercise in the aging population and those suffering from neurodegenerative diseases, have attributed the benefits of physical exercise to increased cardiac output, leading to elevated cerebral blood flow, driving angiogenesis in regions involved in cognition and motor function (Ahlskog, 2011; Paillard et al., 2015; Zigmond et al., 2012). We therefore examined the level of vascularization in the host and graft. Staining for rat endothelial cell antigen-1 (RECA1) revealed a significantly denser vascular network within the host midbrain, compared to the striatum ($p = 0.0062$), a finding that may explain the larger grafts with increased total NeuN+ neurons at this site (Figure 5A). Only in the host striatum did exercise effect angiogenesis, significantly increasing vessel density (Figures 5A and 5C–5F). This effect of exercise was similarly seen in ectopic striatal, but not homotopic midbrain, grafts (Figures 5B–5F).

Exercise drives trophic signaling in the host brain

Finally, we investigated the mechanisms underpinning plasticity changes. Former studies, analysing the intact brain, have highlighted changes in several trophic proteins in response to exercise including brain-derived neurotrophic factor (BDNF) and glial cell-derived neurotrophic factor (GDNF) (reviews: da Silva et al., 2016; Nithianantharajah and Hannan, 2006; van Praag et al., 2000). Following exercise, in lesioned ungrafted animals, we confirmed upregulation of striatal GDNF and BDNF protein levels by immunoblotting (Figures 6A, 6B, and 6F).

Validating striatal GDNF and BDNF upregulation, we assessed the GDNF/GRFa1/RET and BDNF/TrkB downstream intracellular MAPK-ERK pathways that drive activation of gene transcription involved in DA survival and plasticity

striatum. Exercise significantly increased GFP+ fiber density from ectopic grafts in the dorsolateral (F, $p = 0.0028$), dorsomedial (G, $p = 0.0133$), ventrolateral (H, $p = 0.0039$), and ventromedial (I, $p = 0.0011$) striatum. In contrast, significantly lower GFP^+ fiber density was observed from homotopic grafts across all striatal regions, with exercise-induced impacts only seen in the ventromedial striatum ($p = 0.0038$) (I). Further quantification of GFP+ fibers revealed exercise-enhanced fiber density from ectopic grafts that was maintained across the rostral-caudal axis (−0.8 to 1.70 mm) of the dorsolateral (J) and ventrolateral (K) striatum. Regression analysis showed increased GFP+ fiber densities correlated with distance ran/animal (L, $r^2 = 0.66$). Abbreviations: DA, dopamine; Ex, exercise; Tx, transplant. Data are Mean ± SEM. $^*p < 0.05$, $^{**}p < 0.01$, $^{***}p < 0.001$, $^{****}p < 0.0001$ vs. ectopic. $n = 4$/group. Scale bar: 1 mm (A–D) and 200 μm (Ai–Dii).

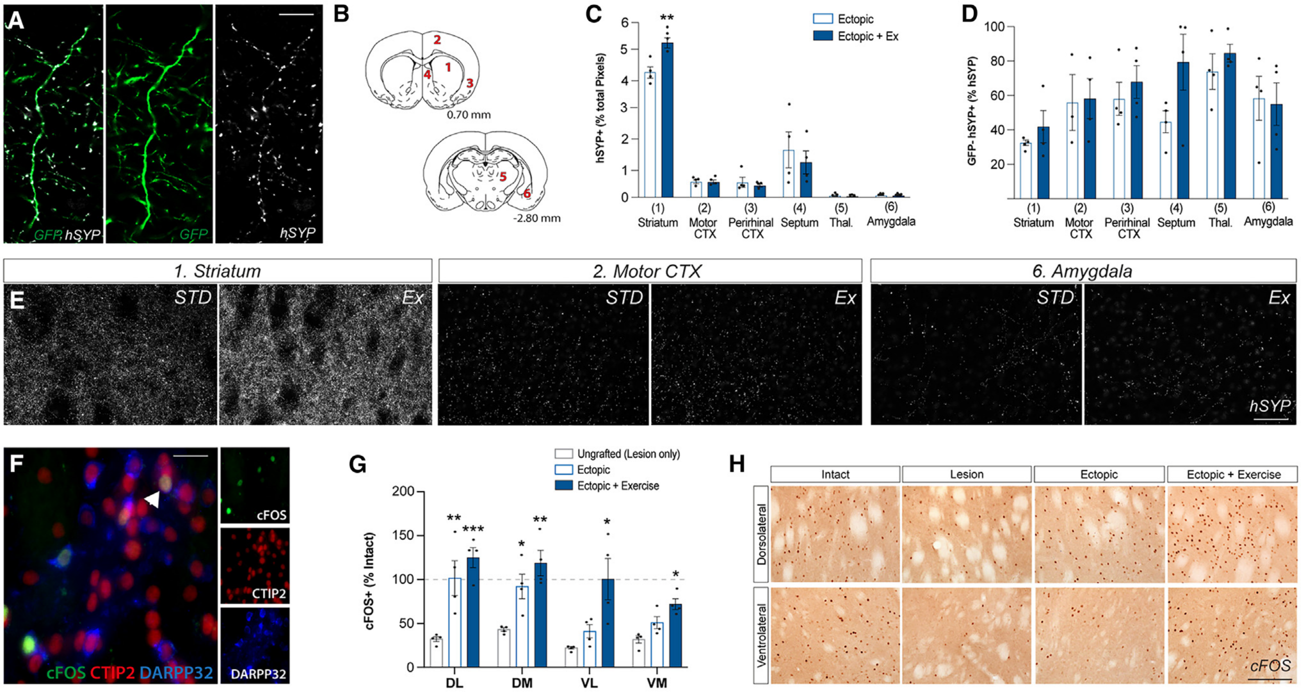

Figure 4. Exercise enhances the integration of transplanted DA neurons

hSYP and GFP colocalization in the striatum indicates graft-derived DA synapse formation (A). Sampling sites for quantification of hSYP+ puncta in the striatum (1), motor cortex (2), perirhinal cortex (3), septum (4), thalamus (5), and amygdala (6) (B). No significant changes in hSYP+ puncta within extrastriatal targets were observed in response to exercise (C). Analysis of graft-derived non-DA inputs (GFP-hSYP+) revealed no exercise-driven changes to striatal or extrastriatal innervation patterns (D). Photomicrographs of hSYP+ puncta in the striatum, motor cortex, and amygdala of animals under standard or exercise conditions (E). cFOS+/CTIP2+/DARPP32+ co-labeling confirmed striatal neuron functional activity (F). Quantification of striatal subregions revealed ectopic grafts ± exercise significantly increased cFOS+ density in the dorsolateral ($p = 0.0021$) and dorsomedial ($p = 0.0042$) striatum, where only exercise resulted in increased cFOS+ density in the ventrolateral ($p = 0.0093$) and ventromedial ($p = 0.0029$) striatum (G). cFOS+ activation in the dorsolateral and ventrolateral striatum (H). Abbreviations: CTX, cortex; DA, dopamine; Ex, exercise; DM, dorsomedial; DL, dorsolateral; STD, standard; VM, ventromedial; VL, ventrolateral. Data are Mean ± SEM. $^*p < 0.05$, $^{**}p < 0.01$, $^{***}p < 0.001$, $^{****}p < 0.0001$ vs. ectopic. $n = 4$/group. Scale bar: 40 μm (A and F) and 200 μm (E and H).

(Kramer and Liss, 2015). Phosphorylated ERK (pERK) was significantly upregulated in the striatum of animals undergoing exercise (Figures 6D, 6E, and 6F). pERK staining enabled detailed assessment of the impact of elevated GDNF levels in both the host MSNs and DA neurons in ectopic grafts. Assessment of the host striatum, ipsi- and contralateral to the lesion, showed upregulation (Intact: 2.39-fold; Lesioned: 2.41-fold) of $pERK^+$ cells in exercised animals (Figures 6G and 6I). Within the graft, 2.2-fold more GFP+ DA neurons expressed pERK, indicative of BDNF/GDNF responsiveness, in animals exposed to exercise (Exercise: 12.06% ± 1.47%; Standard: 5.58% ± 0.55%) (Figures 6H and 6J).

Considering the magnitude of GDNF upregulation (2.7-fold), compared to BDNF (1.5-fold), in animals undergoing exercise, combined with previous reports highlighting GDNF benefits on grafts (Gantner et al., 2020b; Law et al., 2023; Moriarty et al., 2022a; Sinclair et al., 1996), we verified the role of GDNF in exercise-driven functional plasticity by grafting fetal VM from TH-GFP donor mice into GDNF-deficient mice (GDNF knockout [KO]) (Figure S5). Mirroring results seen in ectopic grafts in rats (Figure 2), exercise had no impact on the number of GFP+ cells (Figure S5I) yet significantly increased the volume (Figure S5J) and density (Figure S5K) of GFP+ innervation in GDNF wild-type mice (GDNF WT), highlighting that exercise-driven changes to plasticity hold benefit across not only species (mice vs. rats) but also donor source (hPSC vs. fetal-derived). In contrast, fetal-derived DA cells within GDNF KO mice showed significantly poorer survival (GFP cell counts) and integration (volume and density of striatal GFP innervation), which could not be enhanced by exercise (Figures S5I–S5K), supporting the role of GDNF signaling in exercise-driven plasticity changes.

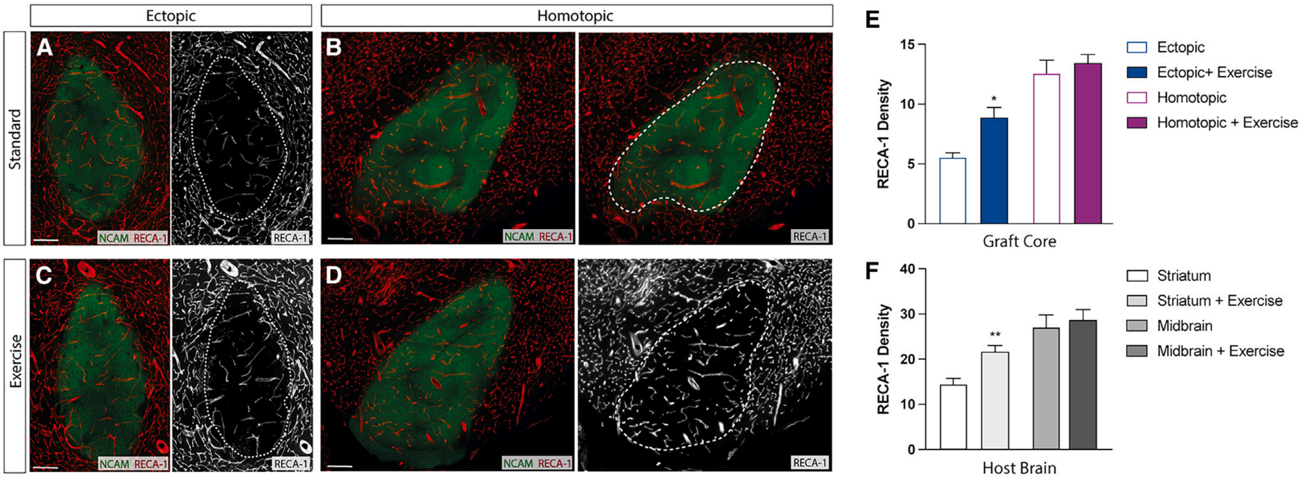

Figure 5. Exercise promotes angiogenesis in both the host striatum and ectopic nerual grafts
Representative images of RECA1 immunolabelling reveals host-derived vasculature within a human NCAM-labeled ectopic (A and C) and homotopic (B and D) graft, highlighting the impact of exercise on an ectopic, but not homotopic graft (E). Similarly, only host striatal, and not midbrain, tissue showed elevated vascularization in response to exercise (A–D and F). Abbreviations: RECA1, rat endothelial cell antigen-1; NCAM, neural cell adhesion molecule. Data are Mean ± SEM. *$p < 0.05$; **$p < 0.01$. n = 5–7/group. Scale bar: 200 μm (C–F).

DISCUSSION

Necessary to achieving optimal functional benefit of neural transplants in the treatment of the motor symptoms in PD is the survival and integration of the new DA neurons. Here we report the benefit of voluntary exercise in improving maturation and plasticity of ectopic placed DA neurons. We show robust demonstration of activity-dependent plasticity, whereby the grafts can change structure and function in response to a changing environment, with exercise selectively promoting the reinnervation of nuclei associated with motor circuits, without impacting the innervation of non-DA targets. Mechanisms of plasticity likely reflect both homeostatic plasticity, that ensures adequate synaptic input, (as would be required in the DA depleted model adopted here and akin to extensive DA innervation loss in PD patients) as well as Hebbian plasticity that sees neurons redistribute synaptic strength to favor the wiring of highly active pathways (Song et al., 2000; Turrigiano and Nelson, 2004). Complementary to the exercise-driven synaptic plasticity in nuclei associated with motor circuitry, was increased response of postsynaptic striatal neurons, seen as striking increases in cFos expressing medium spiny neurons.

Within the midbrain, two major DA neuron populations reside – A9 neurons of the substantia nigra *pars compacta* that are involved in the control of motor function, and A10 neurons within the ventral tegmental area that modulate motivational behaviors. Evidently, restoration of motor function following grafting requires replacement of A9 neurons, with function unable to be restored by other DA neurons, including A10 population (Grealish et al., 2010). We recently showed the necessity of target acquisition in driving terminal maturation of DA neurons, dictating whether DA neurons adopt an A9 or A10 fate (Moriarty et al., 2022b). Correspondingly, in the present findings, the increased striatal reinnervation by grafted DA neurons observed in exercised animals resulted in a significant increase in A9-like GIRK2-expressing DA neurons. The consequence of these activity-dependent changes was a significant improvement in not only induced motor tasks, but spontaneous motor tasks (cylinder and adjusted stepping), not achieved in animals receiving ectopic grafts alone.

An increase in the level of numerous trophic proteins underpin the benefits of exercise in promoting neuronal survival and plasticity through the release of neurotransmitters, including DA (da Silva et al., 2016; Nithianantharajah and Hannan, 2006). Of relevance, Bastioli et al. showed exercise elevated BDNF levels in the intact brain, resulting in increased DA release in the dorsolateral striatum (Bastioli et al., 2022). This has since shown to restore dendritic spine density in MSNs and recovery of corticostriatal long-term potentiation (Marino et al., 2023). Here we show elevated levels of striatal GDNF and BDNF in animals undergoing wheel running. Reflective of elevated levels of these proteins, this resulted in a significant increase in the proportion of host MSNs as well as in graft-derived DA neurons expressing pERK, the intracellular signaling pathway involved in survival and/or plasticity (Kramer and Liss, 2015). Further evidence of the role of GDNF signaling in underpinning the benefits of exercise was shown by the lack of plasticity of grafted DA neurons

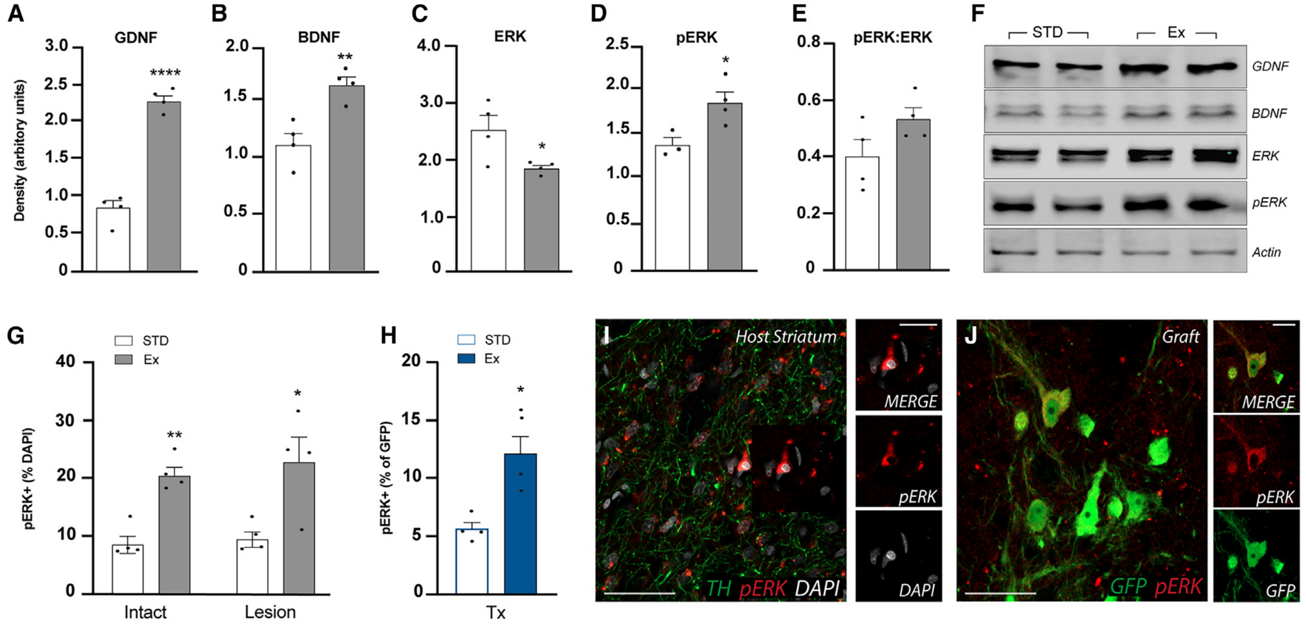

Figure 6. Exercise drives upregulation of trophic signaling in the 6OHDA lesioned striatum
Immunoblotting reveals elevated GDNF (A, $p < 0.0001$) and BDNF (B, $p = 0.0055$) in the host striatum in response to exercise. Validating GDNF and BDNF signaling, exercise decreased total ERK (C, $p = 0.0410$) and increased pERK density (D, $p = 0.0302$), increasing the pERK:ERK ratio (E, $p = 0.116$) — markers of downstream intracellular MAPK-ERK signaling. Representative immunoblots show exercise-induced changes in striatal GDNF (28 kDA), BDNF (14 kDA), ERK (44/42 kDA), and pERK (44/42 kDA) (F). pERK immunohistochemical labeling allowed further validation of downstream signaling. Quantification revealed an exercise-induced upregulation of pERK+ cells within the intact and lesioned striatum in the absence of a DA graft (intact: $p = 0.0014$, lesioned: $p = 0.0267$, G). Within the graft, there was a significant increase in DA neurons expressing pERK in animals housed under exercise conditions ($p = 0.0063$, H). Representative photomicrographs illustrate pERK co-labeling within the striatum (I*:* DAPI+ pERK+) and grafted dopaminergic neurons (J*:* GFP+ pERK+). Abbreviations: Tx, transplant. Data are Mean ± SEM. *$p < 0.05$, **$p < 0.01$, ***$p < 0.001$, ****$p < 0.0001$ vs. ectopic. $n = 4$/group. Scale bar: 200 μm (I and J) and 100 μm (inserts I and J).

implanted into GDNF-deficient mice. These findings corroborate our recent work highlighting the benefit of intrastriatal viral delivery of GDNF to promote the survival and plasticity of DA neurons in both ectopic (striatal placement) and homotopic (VM placed) grafts (Gantner et al., 2020b; Moriarty et al., 2022a).

Interestingly, exercise had little impact on homotopic placed hPSC-derived DA grafts. While graft-derived DA fiber density through the medial forebrain bundle was elevated, these fibers failed to ramify within the target dorsolateral striatum to influence motor recovery. Nonetheless, exercise did increase innervation by homotopic grafts of ventral striatum.

While the focus of the present study was to assess the benefit of exercise on graft plasticity, associated is the possible benefit to the host intrinsic DA system. The potential of neurotrophic factors to exert effects on survival, maturation and plasticity has seen them as promising treatments for a number of degenerative diseases (Aron and Klein, 2011; Bregman et al., 2002; Lindvall and Odin, 1994). However, despite preclinical efficacy, their clinical application via cerebrospinal fluid infusion or viral delivery has been underwhelming due to challenges with trafficking proteins across the blood brain barrier and achieving controlled temporal and spatial delivery.

The current study provides exciting prospects for improving neural grafting. Such observations encourage further investigation, for example addressing if exercise is equally beneficial in older verses younger recipients, the outcomes in males verses females (noting differences in exercise outcomes in pre-versus post-menopausal women (Erickson and Kramer, 2009; Hotting and Roder, 2013)) and of course assessment of exercise intensity and duration. On this later point, while we have evidence that involuntary exercise may hinder benefits due to stress negatively impacting brain chemistry and plasticity (Howells et al., 2005; McEwen and Morrison, 2013; Moraska et al., 2000), the jury remains out regarding optimal duration and exercise type (Hotting and Roder, 2013) and reports not only the potential benefit of differing forms of physical exercise (alone and in combination), but also the potential for virtual reality training, with stroke patients

reporting benefits when computer software was adopted to enable an individual imagine making movements (Page et al., 2009). In addition to these study design variables will be further readouts of the grafting paradigm. Of particular interest will be future studies adopting single cell transcriptomics (as recently performed for hPSC-derived VM grafts (Rajova et al., 2023; Tiklova et al., 2020) and performed in animals undergoing voluntary exercise in the absence of grafts (Methi et al., 2024)), to shed new light on exercise-induced changes within both the grafted cells as well as the surrounding host that may further explain mechanisms of action underpinning exercise benefits, and in the context of the present work, uncover the differences in ectopic and homotopic transplant outcomes.

Exercise presents a non-invasive way to sustain trophic delivery for the benefit of the host, as has been widely reported in preclinical and clinical studies (systematic reviews: (da Silva et al., 2016; Nithianantharajah and Hannan, 2006; van Praag et al., 2000) and now reported for hPSC-derived grafts in the PD context. As such, these findings are of critical and timely importance for clinical translation. Inclusion of exercise to slow disease progression has already been approved given to its safety, lack of side effects and benefit – Cochrane review for exercise in PD patients (Ernst et al., 2023; Folkerts et al., 2023). Yet alone this strategy remains insufficient and highlights needs for combined therapies. In this regard, as the field has already witnessed the benefits of fetal tissue grafts in PD (Barker et al., 2013), and now eagerly awaits multiple clinical trials aimed as assessing the benefit of hPSC-derived grafting in the treatment of PD (Barker et al., 2015), the feasibility for exercise inclusion in subsequent trials presents a highly feasible and realistic approach.

METHODS

Ethics statement: All animal procedures were conducted in agreement with the Australian National Health and Medical Research Council's published Code of Practice for the Use of Animals in Research, and approval granted by The Florey Institute Animal Ethics committee.

Donor cell preparations

Human PSCs: The hiPSC line RM3.5 (passage 33–37), engineered to express GFP under the PITX3 promoter (referred to as PITX3-GFP [Moriarty et al., 2022b]) was cultured as previously described (de Luzy et al., 2021). The line was karyotypically normal and frequently tested for mycoplasma. Differentiation into VM DA progenitors, suitable for grafting, was conducted as previously described (Gantner et al., 2020a, 2020b). In preparation for grafting, D19 VM progenitors were dissociated using Accutase (STEMCELL Technologies) and resuspended at 100,000 cells/μL in maturation media supplemented with ROCK inhibitor Y27632 (10 μM, Sigma-Aldrich).

Fetal tissue-derived cells: Fetal tissue was isolated from TH-GFP embryos at day 12 of gestation (E12), as previously described (Kauhausen et al., 2013). The final cell preparation was resuspended at 100,000 cells/μL in Hank's buffered salt solution containing 0.1% DNase.

Surgical procedures and behavior

Surgeries were performed on 32 athymic (CBHrnu) nude rats (male and female, 7-9 weeks old) and 16 (10 weeks old) GDNF transgenic (WT and KO) mice. Unilateral ablation of the host midbrain DA system was achieved by injection of 6OHDA into the medial forebrain bundle of rats and substantia nigra of mice as previously described (Gantner et al., 2020c).

At 4 weeks post-lesioning animals received ectopic (intrastriatal) or homotopic (intranigra) grafts of VM progenitors derived from PITX3-GFP hiPSCs (for Rats) or TH-GFP fetal tissue (for mice) at 100,000 cells in 1μL. Ectopic coordinates for mice: 0.5 mm anterior, 2.0 mm lateral relative to bregma, 3.2 mm ventral; and for rats: 0.5 mm anterior, 2.5 mm lateral relative to bregma, 4.0 mm ventral; Homotopic coordinates for rats: 4.3 mm anterior, 1.6 mm lateral relative to bregma, and 7.2 mm ventral (Figure 1A).

Motor deficits were assessed 3 weeks after lesioning using amphetamine-induced rotation, cylinder, and adjusted stepping tests as previously described (Somaa et al., 2017; Moriarty et al., 2022b), with retesting performed at intervals following grafting (Figure 1A).

Animal housing and exercise

Stratification of rats into groups: (1) Ungrafted, (2) Ungrafted + Exercise, (3) Ectopic, (4) Ectopic + Exercise, (5) Homotopic, or (6) Homotopic + Exercise. Rats were housed (n = 4/cage) in individual ventilated cages (Techniplast GR1800 Double decker; 462 × 403 × 404 mm) with a 12 h light and 12 h dark cycle. Post-transplantation, rats were individually housed 5 days/week during their dark cycle ± access to running wheels (33 cm radius). Running was quantified through infra-red counting (Scurry Activity Monitoring, Lafayette Instrument Company).

Mice: Randomly assigned to (1) standard housing (Standard) or (2) running wheel access (Exercise). All mice group housed (n = 4–6/cage). Mice in the Exercise group had access to 4 running wheels in the home cage. Cages were cleaned weekly with care taken to ensure nests were minimally disturbed, a factor known to induce stress.

Tissue processing

Immunocytochemistry of *In vitro* cultures and *in vivo* grafts within rat and mice brains were performed as previously described (de Luzy et al., 2021). Primary antibodies and

dilutions are shown in Table S1. Immunoblotting, to measure GDNF, BDNF, and downstream pERK/ERK signaling in the host brain of animals housed in standard or exercise conditions, was performed as previously described (Wang et al., 2014), using antibodies listed in Table S1.

Microscopy and quantification

Bright-field/Dark-field images were captured using a Leica DM6000 microscope and fluorescent images captured using a Leica DM6000, Zeiss Axio Observer, or Zeiss LSM780 confocal microscope.

OTX2+, FOXA2+, TH+, PITX2+, BARHL1+, and PAX6+ cell proportions were assessed from 20x images to confirm hiPSC-derived VM differentiation specification prior to grafting.

HNA+ immunolabelling delineated the graft core, enabling area measurements across serial sections with graft volume (Figure 2E) calculated as previously described (Moriarty et al., 2022a). *In vivo*, GFP+ cells were counted through the entire graft (1:12 series, Figure 2F) and the fraction of GIRK2+ and CALB+ cells quantified by counting GFP+ cells across 3 immunolabelled graft sections (Figure 2G). The number (and proportion) of HNA+ cells, NeuN+ neurons, SOX9+ astrocytes and CC1+ oligodendrocytes were counted in 3 fields of view (20x) and total numbers estimated using density and graft volume (Figures 2I–2K and S3). RECA1 labeling identified host-derived vessels within the graft and host tissue. Vessel density (% total area covered) was estimated as previously described (Somaa et al., 2017). The proportion of pERK+GFP+ DA neurons was calculated as the proportion of total graft DA neurons (GFP+) across a single section. For striatal quantification, pERK+ cells were expressed as percentage of total DAPI.

GFP+ fiber density (Figures 3F–3I) was measured as previously described (Moriarty et al., 2022a). Measurements were made across 5 serial sections/brain, from 1.7 mm anterior to −0.8 mm posterior to bregma and included striatal sampling within the dorsomedial (ML: −1.8, DV: −3.9), ventromedial (ML: −1.8, DV: −5.1), dorsolateral (ML: −3.8, DV: −3.9) and ventrolateral tier (ML: −4.0, DV: −6.9). cFOS+ cell numbers (Figure 4G) were counted in single fields of view (20x) in defined striatal regions at the aforementioned coordinates. For homotopic grafts, innervation was additionally assessed at a single plane within the striatum (0.7 mm anterior to bregma), from ventral (level of the anterior commissure) to dorsal (7 fields of view, 100 μm apart, depicted in Figure S4C). At each site, GFP+ density in single fields of view (20x) was measured.

Graft-derived hSYP+ density in the host striatum and extrastriatal targets was measured as density in single fields of view (20x, Figure 4C). DA (GFP+hSYP+) and non-DA (GFP-hSYP+) integration was further assessed by colocalization of graft-derived fibers. z stack images were acquired as single fields of view (20x) and analyzed using ImarisColoc software (Figure 4D).

Statistical analysis

All data are presented as mean ± SEM. Statistical tests employed were one-way ANOVA with Tukey's *post hoc* multiple comparison, multiple unpaired t tests, and Student's t tests. Numbers of animals/group are stated in figure legends. Statistical analyses were performed using GraphPad Prism with alpha levels of $p < 0.05$ considered significant (*$p < 0.05$, **$p < 0.01$, ***$p < 0.001$, ****$p < 0.0001$).

RESOURCE AVAILABILITY

Lead contact

Requests for further information and resources should be directed to and will be fulfilled by the lead contact, Prof. Clare L. Parish (cparish@unimelb.edu.au).

Materials availability

hiPSC lines utilized in this study are available from the lead contact with a completed materials transfer agreement.

Data and code availability

This article contains all generated and analyzed datasets. Any additional information required is available from the lead contact upon request. This paper reports no original code.

ACKNOWLEDGMENTS

The authors thank Mong Tien and Brianna Xuereb for technical assistance. C.L.P. was supported by a National Health and Medical Research Council Australia (NHMRC) Senior Research Fellowship (APP1154744) and NHMRC L2 Fellowship (GNT2026395). N.M. was supported by an EH Flack fellowship provided by The Marian and E.H. Flack Trust. This work was funded by NHMRC grants (APP2038892 and APP1102704).

AUTHOR CONTRIBUTIONS

Designing studies, N.M., C.L.P., and L.H.T.; conducting experiments, N.M., T.D.F., C.P.J.H., G.E., J.A.K., C.L.P., and L.H.T.; acquiring data, N.M., T.D.F., C.P.J.H., and J.A.K.; analyzing data, N.M., J.A.K., C.L.P., and L.H.T.; providing reagents, C.L.P. and L.H.T.; writing manuscript, N.M., C.L.P., and L.H.T.

DECLARATION OF INTERESTS

The authors declare no competing interests.

SUPPLEMENTAL INFORMATION

Supplemental information can be found online at https://doi.org/10.1016/j.stemcr.2025.102480.

Received: August 19, 2024
Revised: March 25, 2025
Accepted: March 26, 2025
Published: April 24, 2025

REFERENCES

Ahlskog, J.E. (2011). Does vigorous exercise have a neuroprotective effect in Parkinson disease? Neurology *77*, 288–294. https://doi.org/10.1212/WNL.0b013e318225ab66.

Aron, L., and Klein, R. (2011). Repairing the parkinsonian brain with neurotrophic factors. Trends Neurosci. *34*, 88–100. https://doi.org/10.1016/j.tins.2010.11.001.

Barker, R.A., Studer, L., Cattaneo, E., and Takahashi, J.; G-Force PD consortium (2015). G-Force PD: a global initiative in coordinating stem cell-based dopamine treatments for Parkinson's disease. Nature *1*, 15017.

Barker, R.A., Barrett, J., Mason, S.L., and Björklund, A. (2013). Fetal dopaminergic transplantation trials and the future of neural grafting in Parkinson's disease. Lancet Neurol. *12*, 84–91. https://doi.org/10.1016/S1474-4422(12)70295-8.

Barker, R.A., Parmar, M., Studer, L., and Takahashi, J. (2017). Human Trials of Stem Cell-Derived Dopamine Neurons for Parkinson's Disease: Dawn of a New Era. Cell Stem Cell *21*, 569–573. https://doi.org/10.1016/j.stem.2017.09.014.

Bastioli, G., Arnold, J.C., Mancini, M., Mar, A.C., Gamallo-Lana, B., Saadipour, K., Chao, M.V., and Rice, M.E. (2022). Voluntary Exercise Boosts Striatal Dopamine Release: Evidence for the Necessary and Sufficient Role of BDNF. J. Neurosci. *42*, 4725–4736. https://doi.org/10.1523/JNEUROSCI.2273-21.2022.

Brasted, P.J., Watts, C., Robbins, T.W., and Dunnett, S.B. (1999a). Associative plasticity in striatal transplants. Proc. Natl. Acad. Sci. USA *96*, 10524–10529. https://doi.org/10.1073/pnas.96.18.10524.

Brasted, P.J., Watts, C., Torres, E.M., Robbins, T.W., and Dunnett, S.B. (1999b). Behavioural recovery following striatal transplantation: effects of postoperative training and P-zone volume. Exp. Brain Res. *128*, 535–538. https://doi.org/10.1007/s002210050877.

Brasted, P.J., Watts, C., Torres, E.M., Robbins, T.W., and Dunnett, S.B. (2000). Behavioral recovery after transplantation into a rat model of Huntington's disease: dependence on anatomical connectivity and extensive postoperative training. Behav. Neurosci. *114*, 431–436.

Bregman, B.S., Coumans, J.V., Dai, H.N., Kuhn, P.L., Lynskey, J., McAtee, M., and Sandhu, F. (2002). Transplants and neurotrophic factors increase regeneration and recovery of function after spinal cord injury. Prog. Brain Res. *137*, 257–273.

Cenci, M.A., Kalén, P., Mandel, R.J., Wictorin, K., and Björklund, A. (1992). Dopaminergic transplants normalize amphetamine- and apomorphine-induced Fos expression in the 6-hydroxydopamine-lesioned striatum. Neuroscience *46*, 943–957. https://doi.org/10.1016/0306-4522(92)90196-9.

Chang, J.W., Wachtel, S.R., Young, D., and Kang, U.J. (1999). Biochemical and anatomical characterization of forepaw adjusting steps in rat models of Parkinson's disease: studies on medial forebrain bundle and striatal lesions. Neuroscience *88*, 617–628.

da Silva, P.G.C., Domingues, D.D., de Carvalho, L.A., Allodi, S., and Correa, C.L. (2016). Neurotrophic factors in Parkinson's disease are regulated by exercise: Evidence-based practice. J. Neurol. Sci. *363*, 5–15. https://doi.org/10.1016/j.jns.2016.02.017.

de Laat, B., Hoye, J., Stanley, G., Hespeler, M., Ligi, J., Mohan, V., Wooten, D.W., Zhang, X., Nguyen, T.D., Key, J., et al. (2024). Intense exercise increases dopamine transporter and neuromelanin concentrations in the substantia nigra in Parkinson's disease. NPJ Parkinsons Dis. *10*, 34. https://doi.org/10.1038/s41531-024-00641-1.

de Luzy, I.R., Law, K.C.L., Moriarty, N., Hunt, C.P.J., Durnall, J.C., Thompson, L.H., Nagy, A., and Parish, C.L. (2021). Human stem cells harboring a suicide gene improve the safety and standardisation of neural transplants in Parkinsonian rats. Nat. Commun. *12*, 3275. https://doi.org/10.1038/s41467-021-23125-9.

Dobrossy, M.D., Le Moal, M., Montaron, M.F., and Abrous, N. (2000). Influence of environment on the efficacy of intrastriatal dopaminergic grafts. Exp. Neurol. *165*, 172–183. https://doi.org/10.1006/exnr.2000.7462.

Erickson, K.I., and Kramer, A.F. (2009). Aerobic exercise effects on cognitive and neural plasticity in older adults. Br. J. Sports Med. *43*, 22–24. https://doi.org/10.1136/bjsm.2008.052498.

Ernst, M., Folkerts, A.K., Gollan, R., Lieker, E., Caro-Valenzuela, J., Adams, A., Cryns, N., Monsef, I., Dresen, A., Roheger, M., et al. (2023). Physical exercise for people with Parkinson's disease: a systematic review and network meta-analysis. Cochrane Database Syst. Rev. *1*, CD013856. https://doi.org/10.1002/14651858.CD013856.pub2.

Folkerts, A.K., Nielsen, J., Gollan, R., Lansu, A., Solfronk, D., Monsef, I., Ernst, M., Skoetz, N., Zeuner, K.E., and Kalbe, E. (2023). Physical Exercise as a Potential Treatment for Fatigue in Parkinson's Disease? A Systematic Review and Meta-Analysis of Pharmacological and Non-Pharmacological Interventions. J. Parkinsons Dis. *13*, 659–679. https://doi.org/10.3233/JPD-225116.

Gantner, C.W., Cota-Coronado, A., Thompson, L.H., and Parish, C.L. (2020a). An Optimized Protocol for the Generation of Midbrain Dopamine Neurons under Defined Conditions. STAR Protoc. *1*, 100065. https://doi.org/10.1016/j.xpro.2020.100065.

Gantner, C.W., de Luzy, I.R., Kauhausen, J.A., Moriarty, N., Niclis, J.C., Bye, C.R., Penna, V., Hunt, C.P.J., Ermine, C.M., Pouton, C.W., et al. (2020b). Viral Delivery of GDNF Promotes Functional Integration of Human Stem Cell Grafts in Parkinson's Disease. Cell Stem Cell *26*, 511–526.e5. https://doi.org/10.1016/j.stem.2020.01.010.

Gantner, C.W., de Luzy, I.R., Kauhausen, J.A., Moriarty, N., Niclis, J.C., Penna, V., Hunt, C.P., Bye, C.R., Ermine, C.M., Pouton, C.W., et al. (2020c). Viral delivery of GDNF promotes functional integration of human stem cell grafts in Parkinson's disase. Cell Stem Cell *26*, 1–15.

Grealish, S., Diguet, E., Kirkeby, A., Mattsson, B., Heuer, A., Bramoulle, Y., Van Camp, N., Perrier, A.L., Hantraye, P., Björklund, A., and Parmar, M. (2014). Human ESC-derived dopamine neurons show similar preclinical efficacy and potency to fetal neurons when grafted in a rat model of Parkinson's disease. Cell Stem Cell *15*, 653–665.

Grealish, S., Jönsson, M.E., Li, M., Kirik, D., Björklund, A., and Thompson, L.H. (2010). The A9 dopamine neuron component in grafts of ventral mesencephalon is an important determinant for recovery of motor function in a rat model of Parkinson's disease. Brain *133*, 482–495.

Hotting, K., and Roder, B. (2013). Beneficial effects of physical exercise on neuroplasticity and cognition. Neurosci. Biobehav. Rev. *37*, 2243–2257. https://doi.org/10.1016/j.neubiorev.2013.04.005.

Howells, F.M., Russell, V.A., Mabandla, M.V., and Kellaway, L.A. (2005). Stress reduces the neuroprotective effect of exercise in a rat model for Parkinson's disease. Behav. Brain Res. *165*, 210–220. https://doi.org/10.1016/j.bbr.2005.06.044.

Hwang, D.H., Shin, H.Y., Kwon, M.J., Choi, J.Y., Ryu, B.Y., and Kim, B.G. (2014). Survival of neural stem cell grafts in the lesioned spinal cord is enhanced by a combination of treadmill locomotor training via insulin-like growth factor-1 signaling. J. Neurosci. *34*, 12788–12800. https://doi.org/10.1523/JNEUROSCI.5359-13.2014.

Johansson, M.E., Cameron, I.G.M., Van der Kolk, N.M., de Vries, N.M., Klimars, E., Toni, I., Bloem, B.R., and Helmich, R.C. (2022). Aerobic Exercise Alters Brain Function and Structure in Parkinson's Disease: A Randomized Controlled Trial. Ann. Neurol. *91*, 203–216. https://doi.org/10.1002/ana.26291.

Kauhausen, J., Thompson, L.H., and Parish, C.L. (2013). Cell intrinsic and extrinsic factors contribute to enhance neural circuit reconstruction following transplantation in Parkinsonian mice. J. Physiol. *591*, 77–91.

Kramer, E.R., and Liss, B. (2015). GDNF-Ret signaling in midbrain dopaminergic neurons and its implication for Parkinson disease. FEBS Lett. *589*, 3760–3772. https://doi.org/10.1016/j.febslet.2015.11.006.

Law, K.C.L., Mahmoudi, N., Zadeh, Z.E., Williams, R.J., Hunt, C.P.J., Nagy, A., Thompson, L.H., Nisbet, D.R., and Parish, C.L. (2023). A Selective, Hydrogel-Based Prodrug Delivery System Efficiently Activates a Suicide Gene to Remove Undifferentiated Human Stem Cells Within Neural Grafts. Adv. Funct. Mater. *33*, 2305771. https://doi.org/10.1002/adfm.202305771.

Lindvall, O., and Odin, P. (1994). Clinical application of cell transplantation and neurotrophic factors in CNS disorders. Curr. Opin. Neurobiol. *4*, 752–757.

Mak, M.K., Wong-Yu, I.S., Shen, X., and Chung, C.L. (2017). Long-term effects of exercise and physical therapy in people with Parkinson disease. Nat. Rev. Neurol. *13*, 689–703. https://doi.org/10.1038/nrneurol.2017.128.

Malczynska-Sims, P., Chalimoniuk, M., Wronski, Z., Marusiak, J., and Sulek, A. (2022). High-intensity interval training modulates inflammatory response in Parkinson's disease. Aging Clin. Exp. Res. *34*, 2165–2176. https://doi.org/10.1007/s40520-022-02153-5.

Mandel, R.J., Brundin, P., and Björklund, A. (1990). The Importance of Graft Placement and Task Complexity for Transplant-Induced Recovery of Simple and Complex Sensorimotor Deficits in Dopamine Denervated Rats. Eur. J. Neurosci. *2*, 888–894.

Marino, G., Campanelli, F., Natale, G., De Carluccio, M., Servillo, F., Ferrari, E., Gardoni, F., Caristo, M.E., Picconi, B., Cardinale, A., et al. (2023). Intensive exercise ameliorates motor and cognitive symptoms in experimental Parkinson's disease restoring striatal synaptic plasticity. Sci. Adv. *9*, eadh1403. https://doi.org/10.1126/sciadv.adh1403.

Mayer, E., Brown, V.J., Dunnett, S.B., and Robbins, T.W. (1992). Striatal graft-associated recovery of a lesion-induced performance deficit in the rat requires learning to use the transplant. Eur. J. Neurosci. *4*, 119–126. https://doi.org/10.1111/j.1460-9568.1992.tb00858.x.

McEwen, B.S., and Morrison, J.H. (2013). The brain on stress: vulnerability and plasticity of the prefrontal cortex over the life course. Neuron *79*, 16–29. https://doi.org/10.1016/j.neuron.2013.06.028.

Methi, A., Islam, M.R., Kaurani, L., Sakib, M.S., Krüger, D.M., Pena, T., Burkhardt, S., Liebetanz, D., and Fischer, A. (2024). A Single-Cell Transcriptomic Analysis of the Mouse Hippocampus After Voluntary Exercise. Mol. Neurobiol. *61*, 5628–5645. https://doi.org/10.1007/s12035-023-03869-9.

Moraska, A., Deak, T., Spencer, R.L., Roth, D., and Fleshner, M. (2000). Treadmill running produces both positive and negative physiological adaptations in Sprague-Dawley rats. Am. J. Physiol. Regul. Integr. Comp. Physiol. *279*, R1321–R1329. https://doi.org/10.1152/ajpregu.2000.279.4.R1321.

Moriarty, N., Gantner, C.W., Hunt, C.P.J., Ermine, C.M., Frausin, S., Viventi, S., Ovchinnikov, D.A., Kirik, D., Parish, C.L., and Thompson, L.H. (2022a). A combined cell and gene therapy approach for homotopic reconstruction of midbrain dopamine pathways using human pluripotent stem cells. Cell Stem Cell *29*, 434–448.e5. https://doi.org/10.1016/j.stem.2022.01.013.

Moriarty, N., Kauhausen, J.A., Pavan, C., Hunt, C.P.J., de Luzy, I.R., Penna, V., Ermine, C.M., Thompson, L.H., and Parish, C.L. (2022b). Understanding the Influence of Target Acquisition on Survival, Integration, and Phenotypic Maturation of Dopamine Neurons within Stem Cell-Derived Neural Grafts in a Parkinson's Disease Model. J. Neurosci. *42*, 4995–5006. https://doi.org/10.1523/JNEUROSCI.2431-21.2022.

Niclis, J.C., Gantner, C.W., Alsanie, W.F., McDougall, S.J., Bye, C.R., Elefanty, A.G., Stanley, E.G., Haynes, J.M., Pouton, C.W., Thompson, L.H., and Parish, C.L. (2017). Efficiently Specified Ventral Midbrain Dopamine Neurons from Human Pluripotent Stem Cells Under Xeno-Free Conditions Restore Motor Deficits in Parkinsonian Rodents. Stem Cells Transl. Med. *6*, 937–948.

Nithianantharajah, J., and Hannan, A.J. (2006). Enriched environments, experience-dependent plasticity and disorders of the nervous system. Nat. Rev. Neurosci. *7*, 697–709.

Page, S.J., Szaflarski, J.P., Eliassen, J.C., Pan, H., and Cramer, S.C. (2009). Cortical plasticity following motor skill learning during mental practice in stroke. Neurorehabil. Neural Repair *23*, 382–388. https://doi.org/10.1177/1545968308326427.

Paillard, T., Rolland, Y., and de Souto Barreto, P. (2015). Protective Effects of Physical Exercise in Alzheimer's Disease and Parkinson's Disease: A Narrative Review. J. Clin. Neurol. *11*, 212–219. https://doi.org/10.3988/jcn.2015.11.3.212.

Piccini, P., Pavese, N., Hagell, P., Reimer, J., Björklund, A., Oertel, W.H., Quinn, N.P., Brooks, D.J., and Lindvall, O. (2005). Factors affecting the clinical outcome after neural transplantation in Parkinson's disease. Brain *128*, 2977–2986. https://doi.org/10.1093/brain/awh649.

Rajova, J., Davidsson, M., Avallone, M., Hartnor, M., Aldrin-Kirk, P., Cardoso, T., Nolbrant, S., Mollbrink, A., Storm, P., Heuer, A., et al. (2023). Deconvolution of spatial sequencing provides accurate characterization of hESC-derived DA transplants in vivo.

Mol Ther Methods Clin Dev *29*, 381–394. https://doi.org/10.1016/j.omtm.2023.04.008.

Sinclair, S.R., Svendsen, C.N., Torres, E.M., Martin, D., Fawcett, J.W., and Dunnett, S.B. (1996). GDNF enhances dopaminergic cell survival and fibre outgrowth in embryonic nigral grafts. Neuroreport *7*, 2547–2552.

Somaa, F.A., Wang, T.Y., Niclis, J.C., Bruggeman, K.F., Kauhausen, J.A., Guo, H., McDougall, S., Williams, R.J., Nisbet, D.R., Thompson, L.H., and Parish, C.L. (2017). Peptide-Based Scaffolds Support Human Cortical Progenitor Graft Integration to Reduce Atrophy and Promote Functional Repair in a Model of Stroke. Cell Rep. *20*, 1964–1977.

Song, S., Miller, K.D., and Abbott, L.F. (2000). Competitive Hebbian learning through spike-timing-dependent synaptic plasticity. Nat. Neurosci. *3*, 919–926. https://doi.org/10.1038/78829.

Tashiro, S., Nishimura, S., Iwai, H., Sugai, K., Zhang, L., Shinozaki, M., Iwanami, A., Toyama, Y., Liu, M., Okano, H., and Nakamura, M. (2016). Functional Recovery from Neural Stem/Progenitor Cell Transplantation Combined with Treadmill Training in Mice with Chronic Spinal Cord Injury. Sci. Rep. *6*, 30898. https://doi.org/10.1038/srep30898.

Tiklova, K., Nolbrant, S., Fiorenzano, A., Bjorklund, A.K., Sharma, Y., Heuer, A., Gillberg, L., Hoban, D.B., Cardoso, T., Adler, A.F., et al. (2020). Single cell transcriptomics identifies stem cell-derived graft composition in a model of Parkinson's disease. Nat. Commun. *11*, 2434. https://doi.org/10.1038/s41467-020-16225-5.

Torikoshi, S., Morizane, A., Shimogawa, T., Samata, B., Miyamoto, S., and Takahashi, J. (2020). Exercise Promotes Neurite Extensions from Grafted Dopaminergic Neurons in the Direction of the Dorsolateral Striatum in Parkinson's Disease Model Rats. J. Parkinsons Dis. *10*, 511–521. https://doi.org/10.3233/JPD-191755.

Toy, W.A., Petzinger, G.M., Leyshon, B.J., Akopian, G.K., Walsh, J.P., Hoffman, M.V., Vučković, M.G., and Jakowec, M.W. (2014). Treadmill exercise reverses dendritic spine loss in direct and indirect striatal medium spiny neurons in the 1-methyl-4-phenyl-1,2,3,6-tetrahydropyridine (MPTP) mouse model of Parkinson's disease. Neurobiol. Dis. *63*, 201–209. https://doi.org/10.1016/j.nbd.2013.11.017.

Turrigiano, G.G., and Nelson, S.B. (2004). Homeostatic plasticity in the developing nervous system. Nat. Rev. Neurosci. *5*, 97–107. https://doi.org/10.1038/nrn1327.

van Praag, H., Kempermann, G., and Gage, F.H. (2000). Neural consequences of environmental enrichment. Nat. Rev. Neurosci. *1*, 191–198. https://doi.org/10.1038/35044558.

Wang, T.Y., Bruggeman, K.A.F., Sheean, R.K., Turner, B.J., Nisbet, D.R., and Parish, C.L. (2014). Characterization of the stability and bio-functionality of tethered proteins on bioengineered scaffolds: implications for stem cell biology and tissue repair. J. Biol. Chem. *289*, 15044–15051.

Wu, R., Guo, Y., Zhang, L., Zheng, H., Chen, X., Li, M., Xing, Q., Huang, W., Su, Z., Zhang, D., et al. (2022). Physical exercise promotes integration of grafted cells and functional recovery in an acute stroke rat model. Stem Cell Rep. *17*, 276–288. https://doi.org/10.1016/j.stemcr.2021.12.006.

Zhou, W., Barkow, J.C., and Freed, C.R. (2017). Running wheel exercise reduces alpha-synuclein aggregation and improves motor and cognitive function in a transgenic mouse model of Parkinson's disease. PLoS One *12*, e0190160. https://doi.org/10.1371/journal.pone.0190160.

Zigmond, M.J., Cameron, J.L., Hoffer, B.J., and Smeyne, R.J. (2012). Neurorestoration by physical exercise: moving forward. Parkinsonism Relat. Disord. *18*, S147–S150. https://doi.org/10.1016/S1353-8020(11)70046-3.

Stem Cell Reports, Volume *20*

Supplemental Information

Exercise promotes the functional integration of human stem cell-derived neural grafts in a rodent model of Parkinson's disease

Niamh Moriarty, Tyra D. Fraser, Cameron P.J. Hunt, Georgia Eleftheriou, Jessica A. Kauhausen, Lachlan H. Thompson, and Clare L. Parish

SUPPLEMENTARY DATA

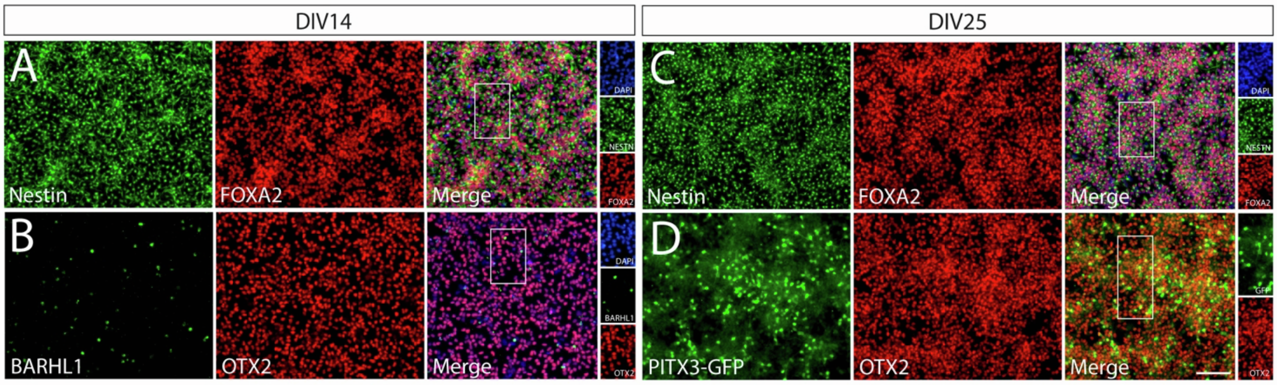

Supplementary Figure 1: Ventral midbrain dopaminergic neurons differentiated from a human induced pluripotent stem cell line expressing enhanced green fluorescent protein under the PITX3 promoter, PITX3-GFP. Photomicrographs showing high FOXA2 and OTX2 expression at D14 (***A,B***). Low numbers of BARHL1+ cells, indicative of off-target rostral progenitors, further validate VM differentiation efficacy (***B***). Ventral midbrain dopamine neuron differentiation was confirmed at D25 by Nestin, FOXA2, PITX3-GFP, and OTX2 expression (***C, D).*** Day (D). Scale bar: 200µm (***A-D***).

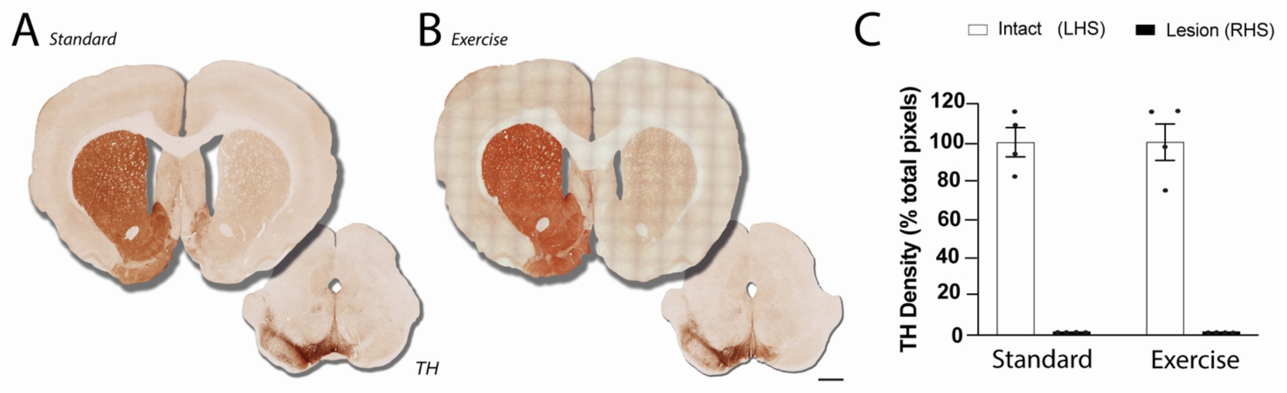

Supplementary Figure 2: Exercise had no impact on the residual host dopaminergic system. Representative images of TH+ immunohistochemistry confirm unilateral 6OHDA lesions in standard (*A*) and exercise (*B*) conditions at 24 weeks. Quantitative analysis of TH+ fibers confirmed that exercise had no impact on the density of dopamine fibers in the host dorsolateral striatum (C). Tyrosine Hydroxylase (TH). Data are Mean ± SEM. n=4/group. Scale bar: 1mm (***A, B***).

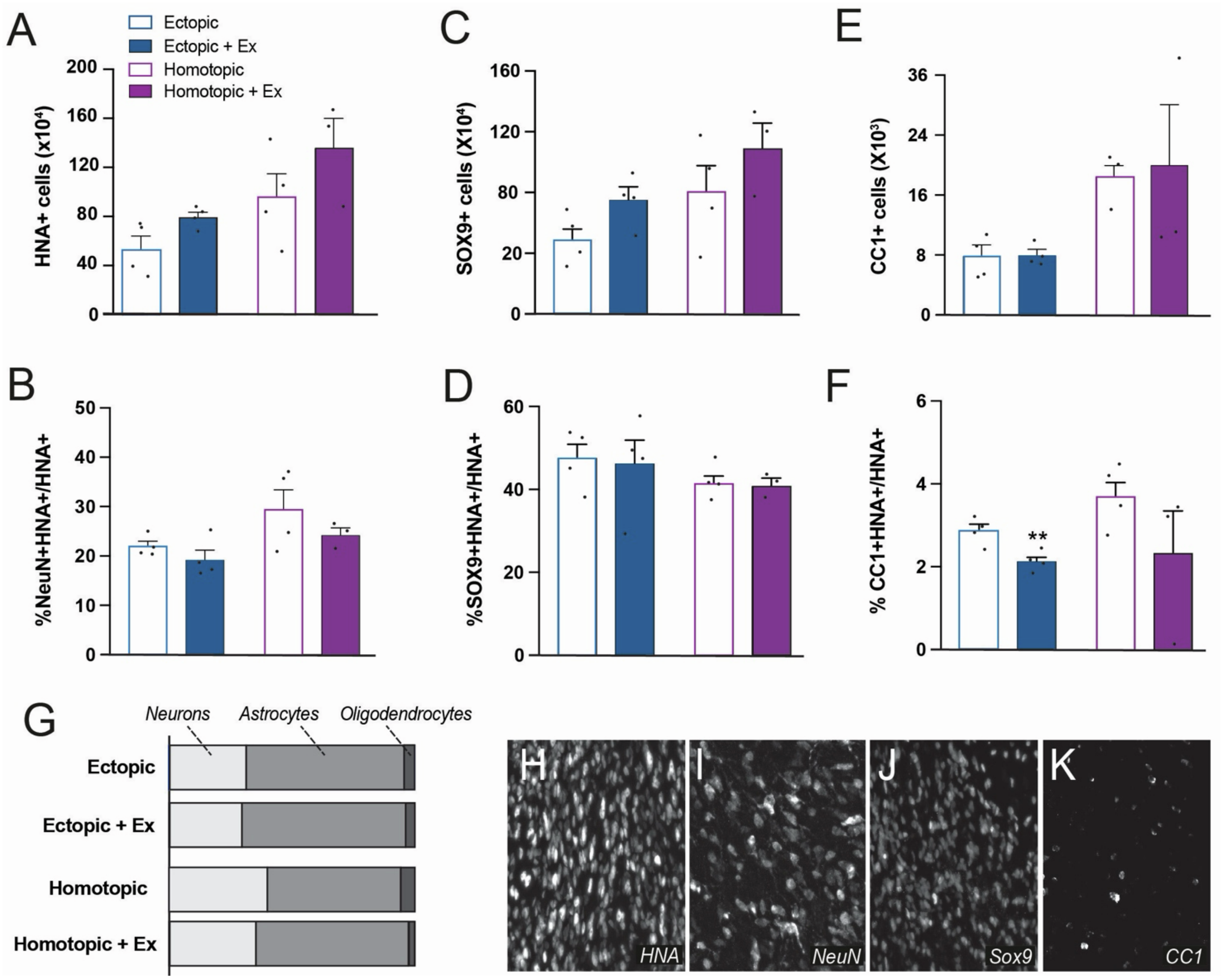

Supplementary Figure 3: Quantification of graft composition revealed that exercise had no significant impact on the total number of HNA+ human cells (**A**), their proportion of NeuN+ neurons (**B**), number or proportion of SOX9+ astrocytes (**C, D**), or CC1+ oligodendrocytes numbers (**E,F**). Only the proportion of CC1+ cells was reduced in ectopic grafts after exercise (**F**). Representation of the proportion of neurons, astrocytes and oligodendrocytes within hPSC-derived grafts (**G**). Representative images depicting HNA (**H**), NeuN (**I**), SOX9 (**J**) and CC1 (**K**) staining within grafts. Human Nuclear Antigen (HNA). Scale bars: 200um (H-K). Data are Mean ± SEM. ***p*<0.01 vs standard. n=4/group.

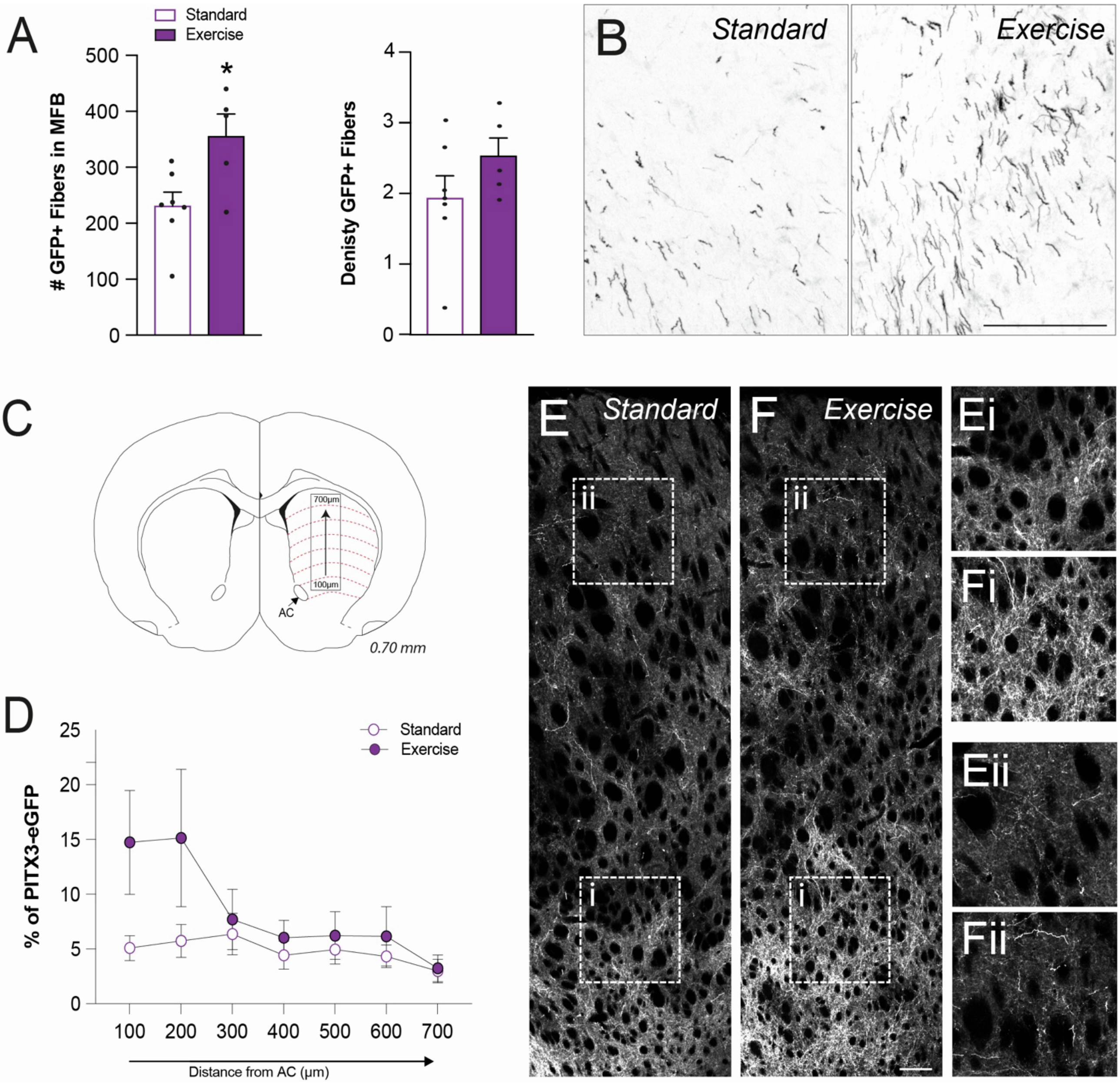

Supplementary Figure 4: Exercise increases dopamine fiber growth along the nigrostriatal pathway. Quantitative analysis revealed a significant increase in the number of fibers transcending through the medial forebrain bundle in animals undergoing an exercise regime (*A*). Photomicrographs showing dopamine fibers in the medial forebrain bundle (*B*). Schematic showing sampling sites along the ventral-dorsal tier of the striatum, 100-700μm from the Anterior Commissure (C). Exercise increased GFP+ fiber density in the ventral striatum, where dopamine fibers emanate from the medial forebrain bundle, however failed to enhance innervation in dorsal tiers (*D*). Representative images showing innervation patterns across the ventral – dorsal striatum under standard (*E*) and exercise (*F*) conditions. High magnification images show GFP+ fibers in the ventral (*Ei, Fi*) and dorsal (*Eii, Fii*) striatum. Anterior Commissure (AC). Data are Mean ± SEM. *$p<0.05$ vs standard. n=5-6/group. Scale bar: 200μm (*B, E, F*).

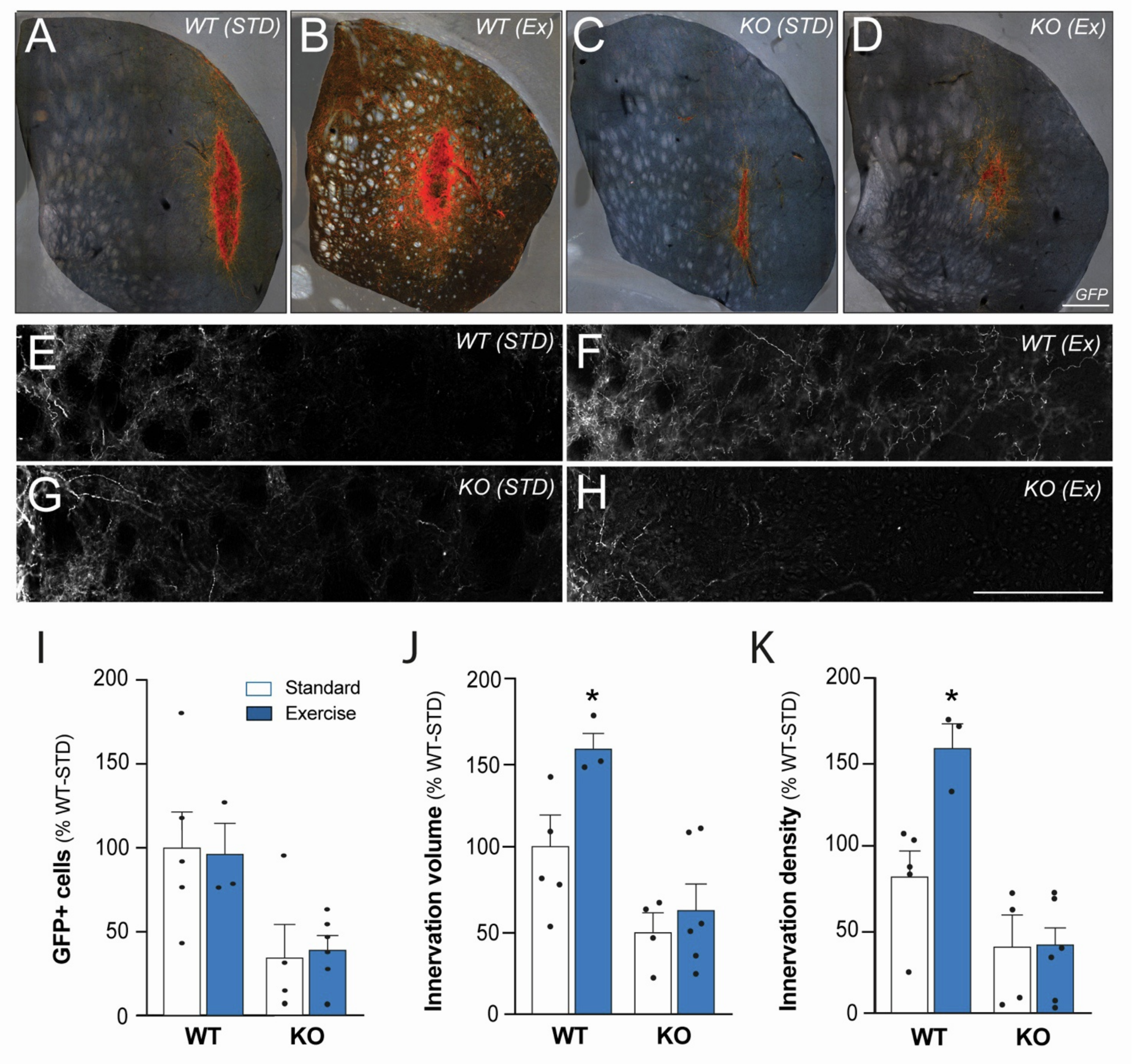

Supplementary Figure 5: Exercise promotes plasticity in WT, but not GDNF-KO mice. Representative images of fetal-derived dopaminergic grafts in the striatum of WT and GDNF-KO mice housed under standard (*A, C, E, F*) or exercise (*B, D, G, H*) conditions. Exercise had no impact on the number of GFP+ cells in WT or GDNF-KO mice (*I*). Exercise has a significant impact on the graft-derived innervation volume (J) and density within the striatum (K) in WT but not KO mice. Exercise (Ex); Knockout (KO); Standard (STD); Wildtype (WT). Data are Mean ± SEM. *p<0.05 vs standard. n=6/group. Scale bar: 1mm (*A-D*).

BARHL1	Rabbit	Novus Biologicals	Cat# NBP1-86513	1:200
BDNF	Rabbit	Abcam	Cat# ab46176	1:2000
Calbindin-C28K	Mouse	Swant	Cat# 300	1:1000
DAPI	-	Sigma Aldrich	Cat# D8417	1:5000
ERK	Rabbit	Cell Signaling Tech	Cat# 91025	1:300
CC1	Mouse	Abcam	Cat# ab16794	1:200
cFOS	Goat	Santa Cruz	Cat# sc-52	1:1000
FOXA2	Goat	Santa Cruz	Cat# sc-6554	1:200
GDNF	Rabbit	Invitrogen	Cat# PA5-89957	1:2000
Green fluorescent protein (GFP)	Chicken	Abcam	Cat# ab13970	1:1000
GFP	Rabbit	Abcam	Cat# ab290	1:1000
GIRK2	Rabbit	Abcam	Cat# ab65096	1:500
Nestin	Mouse	R&D Systems	Cat# MAB353	1:1000
NeuN	Mouse	Millipore	Cat# MAB377	1:1500
OTX2	Goat	R&D Systems	Cat# AF1979	1:500
pERK	Rabbit	Cell Signaling Tech	Cat# A1065	1:300
RECA-1	Mouse	Abd Serotec	Cat# MCA970R	1:2000
SOX9	Rabbit	Abcam	Cat# ab185966	1:500
Human Synaptophysin (hSYP)	Mouse	Enzo Life Sciences	Cat# ADI-905-782-100	1:200
PITX3	Goat	Santa Cruz	Cat# sc-19307	1:200
Tyrosine Hydroxylase (TH)	Rabbit	Pel-freeze	Cat# P40101-0	1:1000
TH	Sheep	Pelfreeze	Cat# P60101-0	1:800

Supplementary Table 1: List of antibodies and dilutions.
